# Hepatocyte membrane potential regulates serum insulin and insulin sensitivity by altering hepatic GABA release

**DOI:** 10.1016/j.celrep.2021.109298

**Published:** 2021-06-29

**Authors:** Caroline E. Geisler, Susma Ghimire, Chelsea Hepler, Kendra E. Miller, Stephanie M. Bruggink, Kyle P. Kentch, Mark R. Higgins, Christopher T. Banek, Jun Yoshino, Samuel Klein, Benjamin J. Renquist

**Affiliations:** 1 School of Animal and Comparative Biomedical Sciences, University of Arizona, Tucson, AZ 85721, USA; 2 Perelman School of Medicine, University of Pennsylvania, Philadelphia, PA 19104, USA; 3 Robert H. Lurie Medical Research Center, Northwestern University, Chicago, IL 60611, USA; 4 Department of Physiology, University of Arizona, Tucson, AZ 85721, USA; 5 Center for Human Nutrition, Washington University School of Medicine, St. Louis, MO, USA; 6 Lead contact

## Abstract

Hepatic lipid accumulation in obesity correlates with the severity of hyperinsulinemia and systemic insulin resistance. Obesity-induced hepatocellular lipid accumulation results in hepatocyte depolarization. We have established that hepatocyte depolarization depresses hepatic afferent vagal nerve firing, increases GABA release from liver slices, and causes hyperinsulinemia. Preventing hepatic GABA release or eliminating the ability of the liver to communicate to the hepatic vagal nerve ameliorates the hyperinsulinemia and insulin resistance associated with diet-induced obesity. In people with obesity, hepatic expression of GABA transporters is associated with glucose infusion and disposal rates during a hyperinsulinemic euglycemic clamp. Single-nucleotide polymorphisms in hepatic GABA re-uptake transporters are associated with an increased incidence of type 2 diabetes mellitus. Herein, we identify GABA as a neuro-hepatokine that is dysregulated in obesity and whose release can be manipulated to mute or exacerbate the glucoregulatory dysfunction common to obesity.

## In brief

Geisler et al. show that obesity depolarizes hepatocytes, favoring transport-mediated GABA release. Hyperpolarizing hepatocytes limits transporter-mediated GABA release. GABA released by the liver decreases vagal afferent nerve activity, causing an increase in serum insulin and insulin resistance. Thus, electrogenic GABA transporters can be modulated to affect glucose homeostasis.

## Graphical Abstract

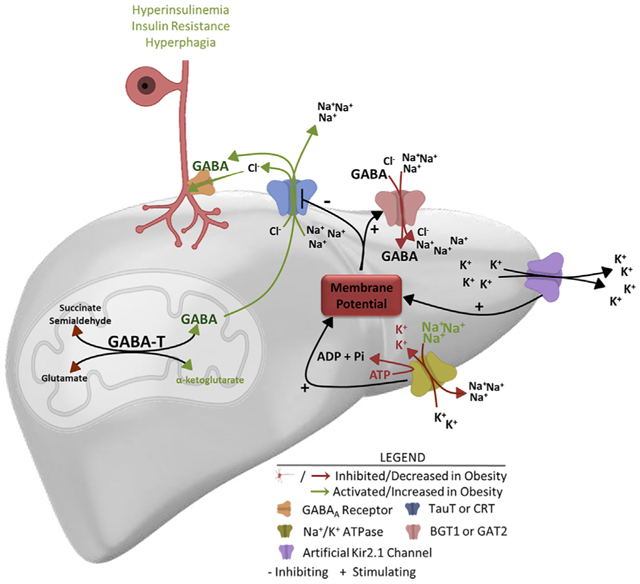

## INTRODUCTION

Non-alcoholic fatty liver disease (NAFLD) is associated with an increased risk of developing diabetes, while the degree of hepatic steatosis is directly related to the severity of systemic insulin resistance, glucose intolerance, and hyperinsulinemia (Chang et al., 2013; Chon et al., 2016; Kotronen et al., 2008). We hypothesize that hepatic lipid accumulation may drive obesity-induced hyperinsulinemia and insulin resistance and aim to identify a potential mechanism by which this could be mediated.

The hepatic vagal afferent nerve (HVAN) communicates with the central nervous system to affect pancreatic insulin release and peripheral tissue insulin sensitivity. A decrease in HVAN firing frequency stimulates insulin secretion, whereas an increase in HVAN firing frequency decreases insulin secretion (Lee and Miller, 1985; Nagase et al., 1993). The HVAN is also involved in regulating whole-body insulin sensitivity. Hepatic vagotomy diminishes insulin sensitivity (assessed as insulin-stimulated glucose uptake) in the insulin-sensitive state, while improving insulin sensitivity and glucose tolerance in a mouse model of insulin resistance (Bernal-Mizrachi et al., 2007; Fernandes et al., 2011). Therefore, the firing frequency of the HVAN can contribute to controlling insulin secretion and sensitivity.

In addition to serving as a hub of metabolism, the liver is also a key endocrine organ that produces a significant number of hepatokines that are altered by obesity, NAFLD, and exercise and signal to change metabolic function in other tissues (Ennequin et al., 2019; Jung et al., 2016; Meex and Watt, 2017; Stefan and Häring, 2013). Despite the established role of the HVAN in affecting both insulin release and sensitivity, none of these hepatokines had been implicated in altering HVAN activity. Hepatocellular lipid accumulation depolarizes hepatocytes (Fitz and Scharschmidt, 1987). Because NAFLD is integral to the development of hyperinsulinemia and insulin resistance, and the HVAN regulates insulin secretion and action, we tested the hypothesis that lipid-induced hepatocyte depolarization changes the release of neurotransmitters to affect firing activity of the HVAN and drive the dysregulation of systemic glucose homeostasis common in obesity.

## RESULTS

### Hepatic vagotomy protects against diet-induced hyperinsulinemia

Chronic hepatic vagotomy eliminates the ability of the liver to alter vagal afferent nerve activity. However, hepatic vagotomy does not prevent otherwise basal signaling of the vagus in the central nervous system (Peters et al., 2013). In fact, basal signaling at the nucleus of the solitary tract (NTS) is restored within 1 month of surgery (Peters et al., 2013).

**Figure 1.**
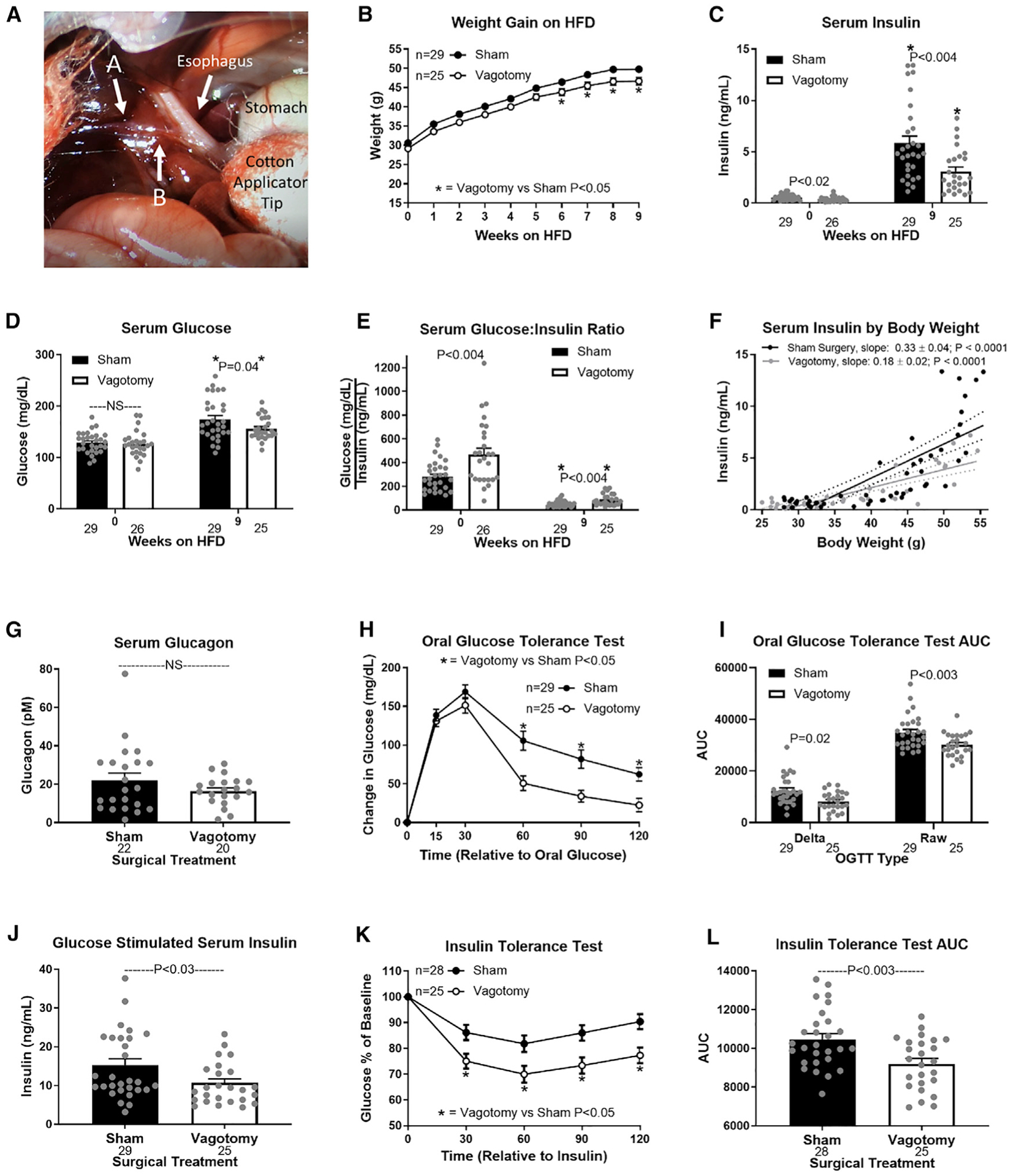
Hepatic vagotomy protects against diet-induced hyperinsulinemia (A) Visual operative field for hepatic vagotomy surgeries. Arrow A indicates the hepatic branch of the vagus, which was severed to vagotomize mice. Arrow A also indicates the electrode placement to record firing activity of the hepatic vagal afferent nerve (Figure 2F). Arrow B indicates where the hepatic vagal nerve was cut after securing the electrode to eliminate vagal efferent activity (Figure 2F). (B–E) Effects of hepatic vagotomy on high-fat-diet (HFD)-induced weight gain (B), serum insulin (C), glucose (D), and glucose/insulin ratio (E) at 0 and 9 weeks. (C–E) Asterisks denote significance (*p < 0.05) between bars of the same color. (F) Regression of body weight and serum insulin concentrations during HFD feeding in sham and vagotomized mice. (G–L) Effect of hepatic vagotomy after 9 weeks of HFD feeding on serum glucagon (G), oral glucose tolerance (OGTT; H), OGTT area under the curve (AUC; I), oral glucose-stimulated serum insulin (J), insulin tolerance (ITT; K), and ITT AUC (L). Number below bar denotes n per group. All data are presented as mean ± SEM. NS, non-significant.

To investigate our hypothesis that hepatic lipid accumulation drives hyperinsulinemia and insulin resistance by altering HVAN activity, we set out to test if obesity-induced insulin dysregulation is dependent on an intact hepatic vagal nerve. We expected that hepatic vagotomy would mute obesity-induced hyperinsulinemia and insulin resistance. We performed hepatic vagotomy or sham surgery in lean mice and then provided them a 60% high-fat diet (HFD; TD 06414; Teklad) for 9 weeks. The operative field is visualized in Figure 1A, with arrow A indicating the hepatic vagal branch that we severed. Weight gain on a HFD was similar between surgical treatments until week 6, after which body weight was lower in hepatic vagotomized mice (Figure 1B). Hepatic vagotomy lowered serum insulin and elevated the glucose/insulin ratio at both 0 and at 9 weeks on the HFD, while decreasing serum glucose concentrations after 9 weeks of HFD feeding (Figures 1C–1E). For the same increase in body weight during HFD feeding, the rise in serum insulin was greater in sham than in vagotomized mice (Figure 1F). Thus, the vagotomy-induced protection against obesity-induced hyperinsulinemia is not due to limited weight gain on the HFD. Importantly, vagotomy did not affect percent fat mass or percent lean mass in chow or 9-week HFD-fed mice (Figures S1B and S1C). Serum glucagon concentrations in HFD-fed mice were not different between surgical treatments (Figure 1G).

Vagotomy improved oral glucose tolerance (OGTT) at 9 weeks on the HFD, while simultaneously decreasing glucose-stimulated insulin concentrations (Figures 1H–1J). Vagotomy also improved insulin sensitivity in obese mice (Figures 1K and 1L). These data support the conclusion that surgically interrupting hepatic vagal signaling attenuates the development of diet-induced hyperinsulinemia and insulin resistance.

### Hepatocyte depolarization depresses HVAN firing activity

**Figure 2.**
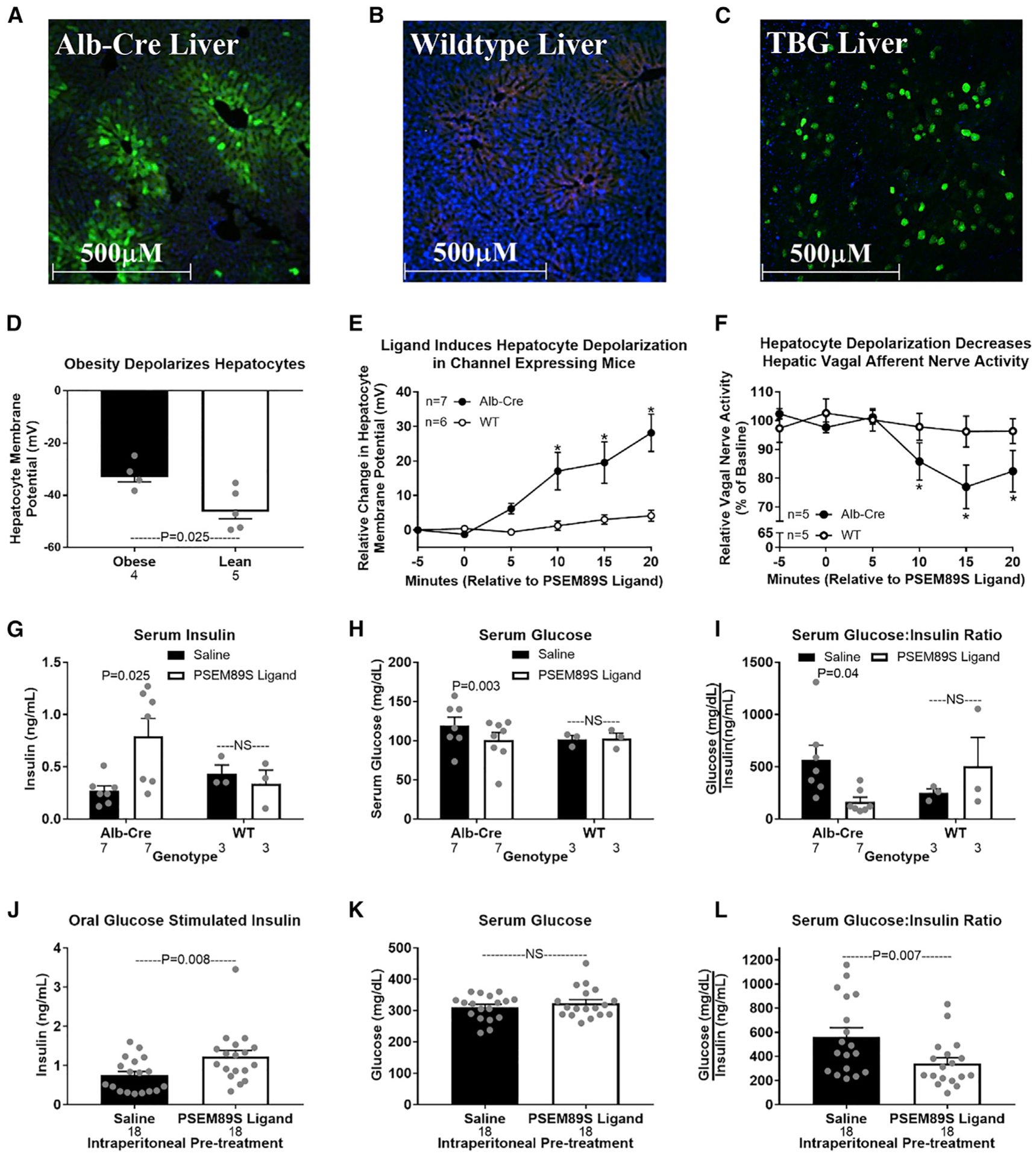
Acute hepatocyte depolarization depresses hepatic vagal afferent nerve activity and elevates serum insulin Immunohistochemical validation of liver-specific viral-induced PSEM89S ligand-gated depolarizing channel. (A–C) Liver from an Alb-Cre-expressing mouse (A) and a WT mouse (B) tail-vein injected with an AAV8 encoding the PSEM89S ligand-activated depolarizing channel and green fluorescent protein (GFP) whose expression is dependent on cre-recombinase. Original magnification, 10×. (D) Liver from a WT mouse tail-vein injected with an AAV8 encoding the PSEM89S ligand-activated depolarizing channel and GFP whose expression is driven by the liver-specific thyroxine binding globulin (TBG) promoter. Green represents GFP, blue represents DAPI (nucleus), and red represents background fluorescence. Hepatocyte membrane potential in lean and obese mice. (E–I) Data from Alb-Cre and WT mice tail-vein injected with an AAV8 encoding liver-specific expression of the PSEM89S ligand-activated depolarizing channel whose expression is dependent on cre-recombinase. PSEM89S ligand (30 μM) induced change in hepatocyte membrane potential (E). PSEM89S ligand induced relative change in hepatic vagal afferent nerve activity (F). Data in (F) were collected concurrently with data in (E). Serum insulin (G), glucose (H), and glucose/insulin ratio (I) in Alb-Cre and WT virus-injected mice 15 min after saline or PSEM89S ligand (30 mg/kg) administration. (J–L) Data from WT mice tail-vein injected with an AAV8 encoding the PSEM89S ligand-activated depolarizing channel whose liver-specific expression is driven by the thyroxine binding globulin (TBG) promoter. Serum insulin (J), glucose (K), and glucose/insulin ratio (L) in channel-expressing mice injected with either saline or PSEM89S ligand (30 mg/kg) 10 min prior to an oral glucose load (2.5 g/kg). Asterisk denotes significance (*p < 0.05) between groups within a time point. Number below bar denotes n per group. All data are presented as mean ± SEM. Alb-Cre, albumin-cre; WT, wild-type.

Obesity depolarizes hepatocytes (Figure 2D). We hypothesize that obesity-induced hepatocyte depolarization is communicated through the HVAN to dysregulate insulin secretion and action. We used the genetically engineered, PSEM89S ligand-gated depolarizing ion channel described by Magnus et al. (2011) to assess the effect of hepatocyte depolarization on HVAN firing activity (Magnus et al., 2011). We intravenously delivered an adeno-associated virus serotype 8 (AAV8) encoding this PSEM89S ligand-gated depolarizing channel and green fluorescent protein (EGFP) flanked by LoxP sites to wild-type mice or mice expressing cre-recombinase driven by the albumin promoter (Michael et al., 2000; Thierbach et al., 2005). This channel will only be expressed in hepatocytes of cre-recombinase-expressing mice and activated by PSEM89S ligand. We performed immunohistochemistry against GFP to confirm liver-specific expression in albumin-cre-expressing mice and no expression in wild-type mice (Figures 2A and 2B). No GFP expression was observed in skeletal muscle, pancreas, or adipose tissue of albumin-cre mice (Figure S2A). To assess the influence of hepatocyte depolarization on HVAN firing, we simultaneously measured hepatocyte membrane potential and HVAN activity in the anesthetized mouse. The hepatic vagal nerve was gently lifted and placed onto a hook-shaped electrode to record HVAN firing activity (Figure 1A, arrow A). After the electrode placement was secured, the hepatic vagal nerve to the right of the hook near the esophagus (Figure 1A, arrow B) was cut to eliminate efferent firing activity. Bath application of the PSEM89S ligand (30 μM) depolarized hepatocytes and decreased HVAN firing activity in albumin-cre, channel-expressing mice (Figures 2E and 2F), while having no effect in wild-type mice (Figures 2E and 2F).

### Acute hepatic depolarization elevates serum insulin

Hepatocyte depolarization depresses HVAN activity (Figures 2E and 2F), while loss of HVAN signaling in obesity protects against the development of hyperinsulinemia (Figure 1C). Therefore, we hypothesized that hepatocyte depolarization caused hyperinsulinemia by altering HVAN activity. To directly test this causal relationship, we intraperitoneally injected PSEM89S ligand (30 mg/kg) to induce hepatocyte depolarization and showed that this more than doubled serum insulin and decreased serum glucose concentrations in albumin-cre, channel-expressing mice (Figures 2G and 2H). Accordingly, PSEM89S ligand decreased the glucose/insulin ratio in albumin-cre mice (Figure 2I). Notably, PSEM89S ligand did not alter serum insulin, glucose, or the glucose/insulin ratio in wild-type mice (Figures 2G–2I).

We developed a second model of hepatocyte depolarization in which liver-specific expression of the same PSEM89S ligand-gated depolarizing channel and GFP was independent of cre-recombinase and instead driven by the liver-specific thyroxine binding globulin (TBG) promoter (Ballantyne et al., 2016; Yan et al., 2012). Wild-type mice intravenously injected with this AAV8 had liver-specific GFP expression confirmed by immunohistochemistry (Figure 2C). No GFP expression was observed in skeletal muscle, pancreas, or adipose tissue (Figure S2B). To further validate that the GFP-positive cells in the liver are hepatocytes, we performed double immunohistochemistry for GFP and the hepatocyte-specific marker, arginase-1 (Figures S2C–S2E). To ensure stimulatory concentrations of circulating glucose, we gave an oral glucose gavage (2.5 g/kg body weight) 10 min following intraperitoneal PSEM89S ligand (30 mg/kg) injection. As previously observed, PSEM89S ligand administration elevated serum insulin and lowered the glucose/insulin ratio in channel-expressing mice (Figures 2J and 2L). PSEM89S ligand injection did not affect the rise in serum glucose following an oral gavage of glucose (Figure 2K). These data establish that acute hepatocyte depolarization depresses HVAN firing activity and increases serum insulin concentrations. With insulin measured at only a single time point measure, we cannot rule out a change in the kinetics of insulin release or clearance.

### Hepatic hyperpolarization protects against diet-induced metabolic dysfunction

**Figure 3.**
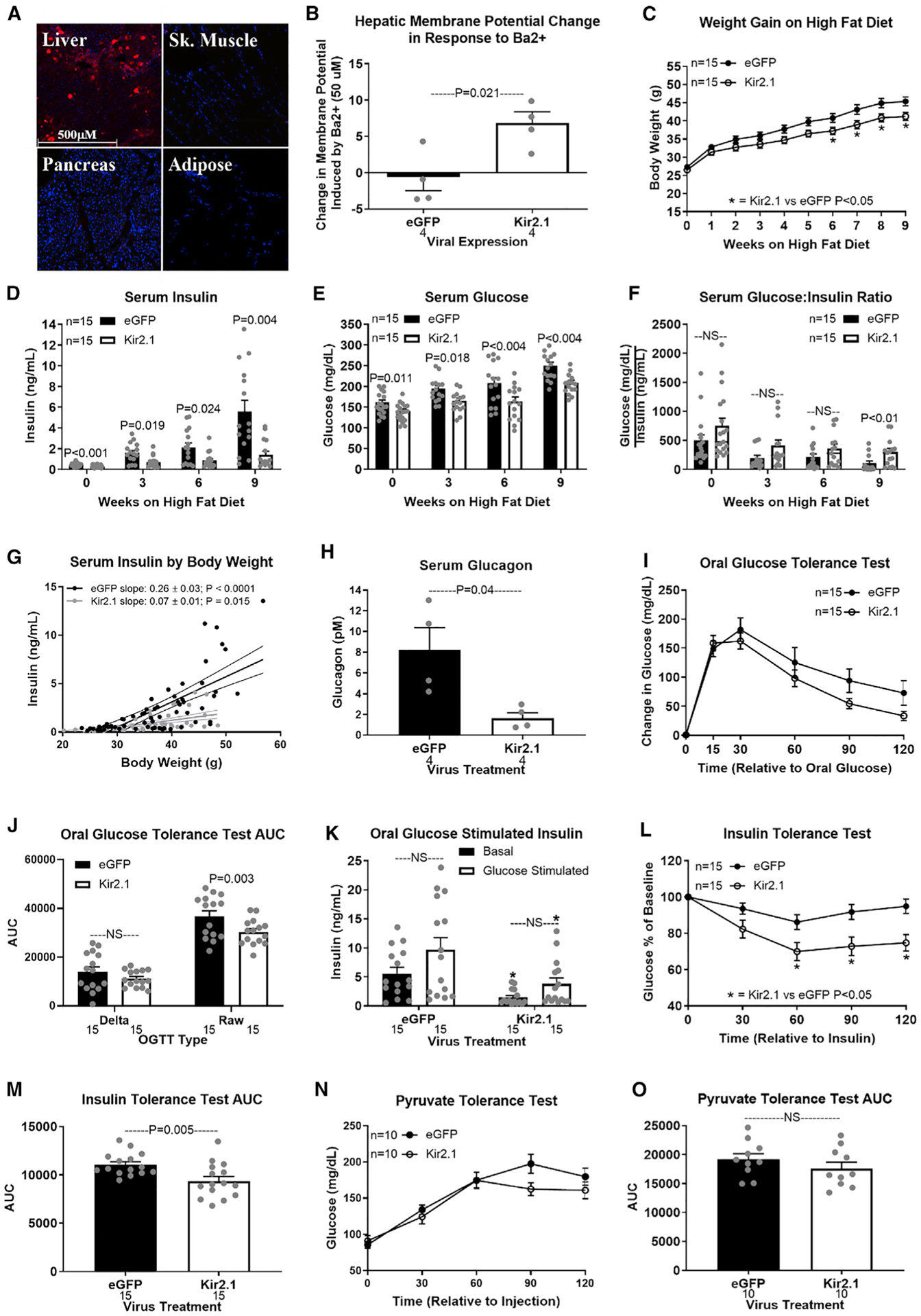
Hepatic hyperpolarization protects against diet-induced metabolic dysfunction (A and B) Liver-specific expression of the Kir2.1 hyperpolarizing channel in a WT mouse (A; original magnification, 10×). Fluorescent imaging for red indicates tdTomato and blue indicates DAPI (nucleus). Barium (BaCl; 50 μM)-induced change in hepatocyte membrane potential in Kir2.1 and EGFP (control)-expressing mice (B). (C–F) Hepatic Kir2.1 expression effect on HFD-induced weight gain (C), serum insulin (D), glucose (E), and glucose/insulin ratio (F) at 0, 3, 6, and 9 weeks. (G) Regression of body weight and serum insulin concentrations during HFD feeding in Kir2.1 and EGFP mice. (H–O) Effect of hepatic Kir2.1 expression after 9 weeks of HFD feeding on serum glucagon (H), oral glucose tolerance (OGTT; I), OGTT AUC (J), oral glucose-stimulated serum insulin (K; asterisk denotes significance [*p < 0.05] between bars of the same color), ITT (L), ITT AUC (M), pyruvate tolerance (PTT; N), and PTT AUC (O). Number below bar denotes n per group. All data are presented as mean ± SEM.

Having established that hepatocyte depolarization increases serum insulin concentrations (Figure 2), and that the hepatic vagus is essential for diet-induced hyperinsulinemia (Figure 1C), we next hypothesized that hepatocyte hyperpolarization would prevent obesity-induced hyperinsulinemia. To induce a chronic hyperpolarized state, we used an AAV8 vector encoding TBG promoter-driven expression of the K^+^ channel, Kir2.1, and EGFP (Figure 3A). Although this channel is inwardly rectifying in neurons, in hepatocytes, with a resting membrane potential that ranges from −20 to −50 mV, Kir2.1 expression supports K^+^ efflux and hyperpolarization (Johns et al., 1999). We confirmed the hyperpolarizing effect of Kir2.1 by *in vivo* intracellular measurement of hepatocyte membrane potential before and after bath application of the Kir2.1 antagonist, barium (Ba^2+^; 50 μM) (Johns et al., 1999). Ba^2+^ induced a 6.86 ± 1.54 mV depolarization of hepatocytes in Kir2.1-expressing mice but had no effect (−0.62 ± 1.86 mV) in control EGFP-expressing mice (Figure 3B).

In lean mice, hepatocyte hyperpolarization decreased basal serum insulin and glucose concentrations (Figures S3A–S3C), improved glucose clearance (Figures S3D–S3F), and enhanced insulin sensitivity (Figures S3G and S3H). Kir2.1 expression did not affect gluconeogenic potential, as assessed by a pyruvate tolerance test (Figures S3I and S3J). This establishes that hepatocyte membrane potential affects systemic glucose homeostasis in non-disease, non-obese conditions.

Kir2.1 expression depressed weight gain on a HFD, reaching significance from weeks 6 to 9 (Figure 3C). At 3 weeks on the HFD, Kir2.1 expression tended to improve insulin sensitivity (p = 0.064; Figures S4D and S4E). Kir2.1 expression limited the rise in serum insulin and glucose in response to 3, 6, or 9 weeks of HFD feeding and increased the glucose/insulin ratio after 9 weeks on the HFD (Figures 3D–3F). Although Kir2.1 expression limited HFD-induced weight gain, at the same body weight serum insulin concentrations were higher in EGFP control than in Kir2.1-expressing mice (Figure 3G). Kir2.1 expression decreased serum glucagon in diet-induced obese mice (Figure 3H). After 9 weeks on the HFD, Kir2.1 expression did not affect glucose clearance (OGTT delta area under the curve [AUC]) or glucose-stimulated serum insulin (Figures 3I–3K). Kir2.1 expression improved insulin sensitivity (Figures 3L and 3M) while having no effect on gluconeogenic potential from pyruvate in diet-induced obese mice (Figures 3N and 3O). These results suggest that hepatocyte hyperpolarization protects against the development of hyperinsulinemia, hyperglucagonemia, hyperglycemia, and insulin resistance in diet-induced obesity.

Importantly, Kir2.1 expression did not affect HFD-induced hepatic lipid accumulation (Kir2.1 versus EGFP control: 94.2 ± 10.6 versus 98.4 ± 6.6 mg triglycerides/g liver; p = 0.73). The absence of hyperinsulinemia in obese Kir2.1-expressing mice despite the development of hepatic steatosis supports hepatocyte depolarization as a critical mediator in the relationship between hepatic lipid accumulation and dysregulated glucose homeostasis.

### Obesity alters hepatocyte neurotransmitter production, while membrane potential affects neurotransmitter release

Resolving the mechanism by which hepatocyte membrane potential can alter HVAN activity and downstream glucose homeostasis is critical to understanding the link between fatty liver and insulin dysregulation. To investigate whether the liver releases neurotransmitters to affect HVAN activity, we incubated liver slices *ex vivo* and measured the release of neurotransmitters into the media (Table S1).

**Figure 4.**
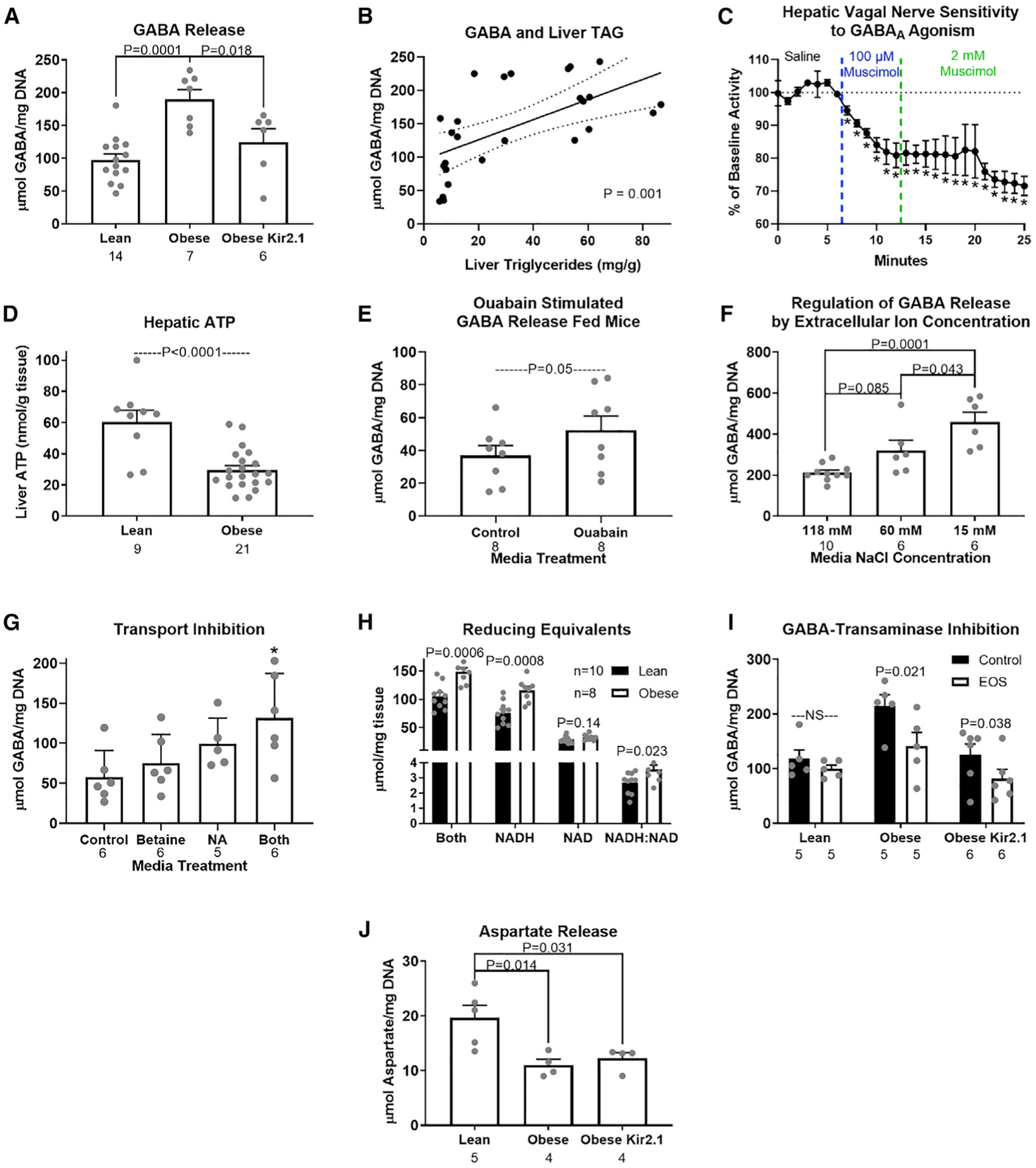
Hepatic slice GABA release (A–J) Release of GABA (μmol/mg DNA) from hepatic slices (A); relationship between hepatic GABA release and liver triglyceride concentration (B); hepatic vagal nerve activity in response to the GABA_A_ receptor agonist, muscimol (C); hepatic ATP concentration (nmol/g tissue; D); release of GABA in slices incubated with the Na^+^/K^+^-ATPase inhibitor, Ouabain (1 mM; E); release of GABA (μmol/mg DNA) from hepatic slices in normal (118 mM), reduced (60 mM), and low (15 mM) NaCl media (F); GABA media concentrations in slices treated with the BGT1 inhibitor (betaine, 1 mM), the GAT2 inhibitor (nipecotic acid [NA], 1 mM), or both (G); reducing equivalent measures from livers of lean and obese mice (H), GABA media concentrations in response to inhibition of GABA-transaminase (EOS, 5.3 mM) in liver slices (I); and aspartate media concentrations in lean, obese, and obese Kir2.1-expressing mice (J). Asterisk indicates difference from control (*p < 0.05). Number below bar denotes n per group. All data are presented as mean ± SEM.

Because obesity depolarizes hepatocytes (Figure 2D), and hepatocyte depolarization decreases HVAN firing activity (Figures 2E and 2F), we hypothesized that livers from obese mice release more of inhibitory or less of excitatory neurotransmitters. Liver slices from obese mice released more of the inhibitory neurotransmitter GABA than liver slices from lean mice (Figure 4A). Hepatocytes synthesize GABA via the mitochondrial enzyme GABA-transaminase (GABA-T) (White, 1981). By measuring liver triglyceride concentration in the same livers from which we had measured media GABA concentrations, we show that with an increase in liver triglyceride concentration there is increased media GABA concentration (Figure 4B). Bath application of the GABA_A_ receptor agonist, muscimol, dose dependently decreased firing activity of the HVAN in the anesthetized mouse (Figure 4C), supporting that hepatocyte-released GABA could serve as the hepatokine signal linking changes in hepatocyte membrane potential and HVAN activity.

**Figure 5.**
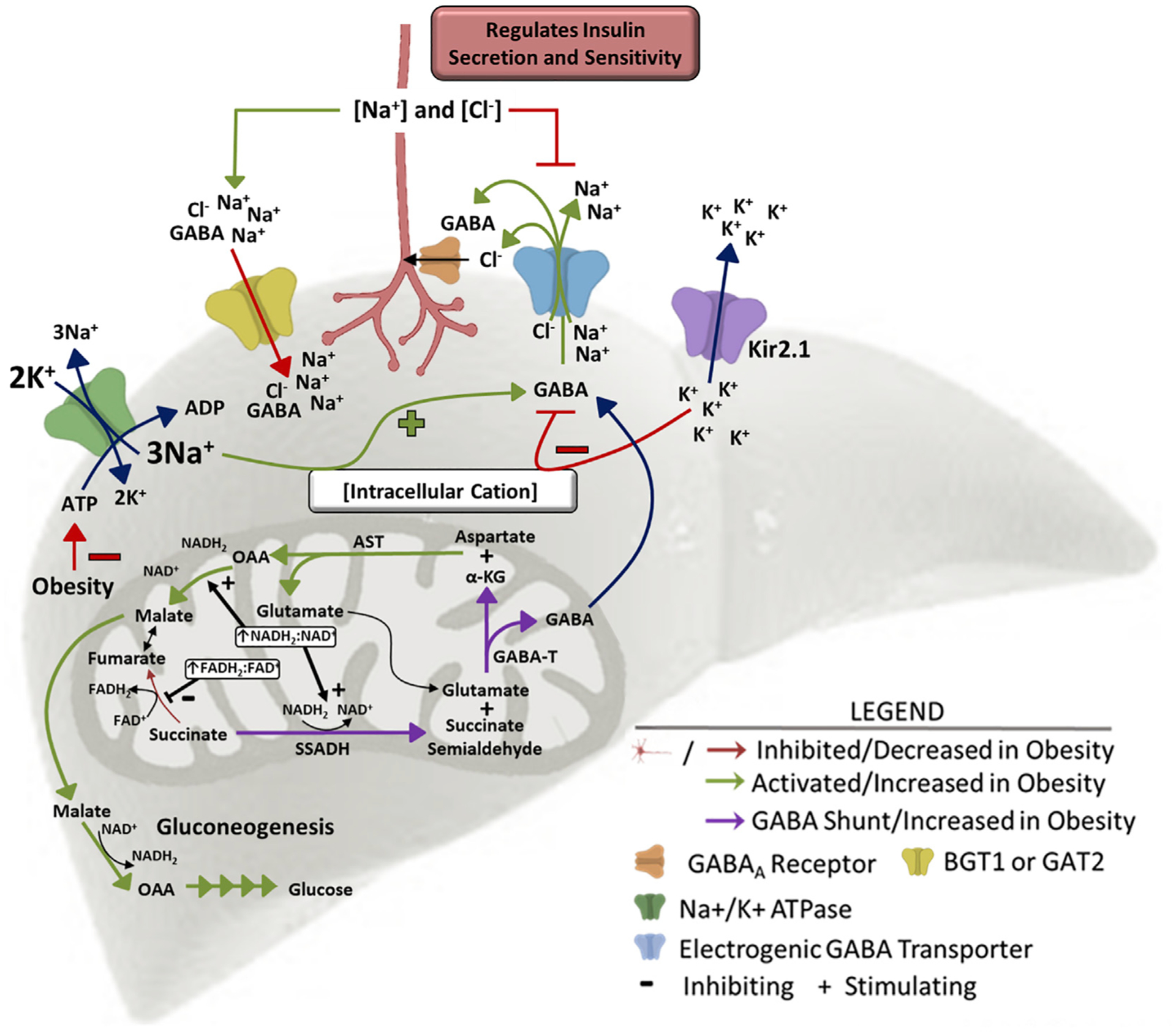
Working model of hepatic lipid accumulation-induced changes in hepatic metabolism and resulting changes in hepatic vagal nerve signaling to affect insulin secretion and sensitivity High levels of β-oxidation in the obese liver increase the mitochondrial NADH_2_:NAD^+^ and FADH_2_:FAD^+^ ratios driving succinate to succinate semialdehyde, generating substrate for GABA-transaminase. GABA-transaminase produces GABA and α-ketoglutarate, a substrate for aspartate aminotransferase. Increased gluconeogenic flux in obesity drives the mitochondrial export of OAA as malate. The increased GABA release is encouraged by the depolarized membrane in obesity. GABA is co-transported with 3 Na^+^ and 1 Cl^−^ ion, so an increase in intracellular cation concentration (hepatocyte depolarization) encourages GABA export, while a decrease in intracellular cation concentration (hepatocyte hyperpolarization) limits GABA export. Kir2.1 expression induces hepatic K^+^ efflux and hyperpolarization, inhibiting GABA export. Obesity decreases hepatic ATP concentrations, impairing activity of the Na^+^/K^+^-ATPase pump and increasing intracellular Na^+^ concentrations, driving GABA export. This mechanism explains how hepatic lipid accumulation increases hepatic GABA release. AST, aspartate aminotransferase; GABA-T, GABA-transaminase; α-KG, α-ketoglutarate; OAA, oxaloacetate; SSADH, succinate semialdehyde dehydrogenase.

Diet-induced obesity decreased hepatic ATP concentrations to less than 50% of that seen in lean mice (Figure 4D) and lowered hepatic activity of the Na^+^/K^+^ ATPase (Stanimirovic et al., 2018). We propose that increased intracellular sodium ions and hepatocyte depolarization resulting from diminished Na^+^/K^+^-ATPase activity in obesity promotes GABA efflux through electrogenic GABA transporters (Figure 5). Pharmacologically inhibiting Na^+^/K^+^-ATPase activity in the liver slice by bath application of Ouabain increased GABA release by more than 40% (Figure 4E). Conversely, Kir2.1 expression, which limited obesity-induced hepatocyte depolarization, decreased obesity-induced hepatocyte slice GABA release (Figure 4A). Having established a key role of membrane potential in the regulation of hepatic slice GABA release and re-uptake, we aimed to better understand hepatic GABA transport.

Given the role of membrane potential in regulating media GABA concentrations, we proposed that liver slice GABA import and export may be mediated by ion-dependent transporters. The liver expresses four electrogenic GABA transporters that are members of the Na^+^/Cl^−^-dependent neurotransmitter transporter (SLC6) family. These include proteins encoded for by Slc6A12 (betaine GABA transporter 1 [BGT1]), Slc6A13 (GABA transporter 2 [GAT2]), Slc6A6 (taurine transporter [TauT]), and Slc6A8 (creatine transporter [CRT]). BGT1 and GAT2 both co-transport 3 Na^+^, 1 Cl^−^, and GABA, moving 2 positive charges in the direction of GABA transport (Eskandari et al., 2017). TauT co-transports 2.5 Na^+^, 1 Cl^−^, and GABA, moving 1.5 positive charges in the direction of GABA transport (Voss et al., 2004). The CRT transporter co-transports 2 Na^+^, 1 Cl^−^, and GABA (or creatine), moving a single positive charge in the direction of GABA transport (ten Hove et al., 2008). To establish the role of these ion-dependent transporters in GABA export, we showed that hepatic slice GABA release was encouraged by incubation in media with low NaCl concentrations (Figure 4F). We also showed that incubation with the BGT1 and GAT2 inhibitors, betaine (1 mM) and nipecotic acid (NA; 1 mM), respectively, increases media GABA concentrations (Figure 4G), establishing their redundant but key role in hepatic GABA re-uptake (Figure 5). We hypothesize that BGT1 and GAT2 may primarily mediate hepatic GABA uptake, while TauT and CTR may primarily mediate hepatic GABA release. Because the liver is a primary sight of creatine production, it is evident that the CTR transporter can work against hepatic membrane potential to export its cargo. Additional studies will be required to fully assess the role of these electrogenic GABA transporters in GABA export. However, our studies establish the key role of membrane potential in regulating activity of these electrogenic GABA transporters results to balance GABA release and re-uptake (Figure 5).

GABA-T is most frequently thought to be an enzyme key to GABA breakdown. In fact, the GABA shunt classically refers to a tricarboxylic acid (TCA) cycle detour that converts α-ketoglutarate to succinate and concomitantly breaks down a molecule of GABA via GABA-T (Hassel et al., 1998). However, early *in vitro* studies established that the reaction in the direction of GABA synthesis was favored with an equilibrium constant (Keq) of 0.04 (van der Laan et al., 1979). The reason that this reaction most frequently proceeds in the reverse direction is a lack of succinate semialdehyde (SSA), for which SSA dehydrogenase (SSADH) has a nearly 10× lower Km (substrate concentration which permits half maximal velocity of the reaction) than GABA-T. In our model (Figure 5), we propose that the elevated NADH:NAD ratio observed in obesity favors the production of SSA and ultimately GABA (Figure 4H). To establish the key role of GABA-T activity in hepatocyte GABA release, we treated liver slices with the irreversible GABA-T inhibitor, ethanolamine-O-sulfate, *ex vivo* (EOS; 5.3 mM). EOS decreased GABA export from obese control and obese Kir2.1-expressing liver slices, but not liver slices from lean mice (Figure 4I). This supports the hypothesis that GABA production is elevated in obesity, and that GABA-T mediated synthesis of GABA is not impaired by Kir2.1 expression. Hepatocytes from obese mice also released less of the excitatory neurotransmitter, aspartate, than hepatocytes from lean mice (Figure 4J). Our model proposes that this decrease in aspartate is a direct effect of the GABA-T-stimulated α-ketoglutarate synthesis and transamination to produce oxaloacetate to support gluconeogenic flux (Figure 5). There was no effect of Kir2.1 expression on the obesity-induced decrease in aspartate release from liver slices (Figure 4J). Because hepatic Kir2.1 expression is able to prevent the hyperinsulinemia and insulin resistance in obesity (Figures 3D and 3L) but does not affect the decrease in hepatic slice aspartate release (Figure 4J), this suggests that a decreased excitatory signal at the HVAN in obesity is not responsible for the development of insulin resistance.

**Figure 6.**
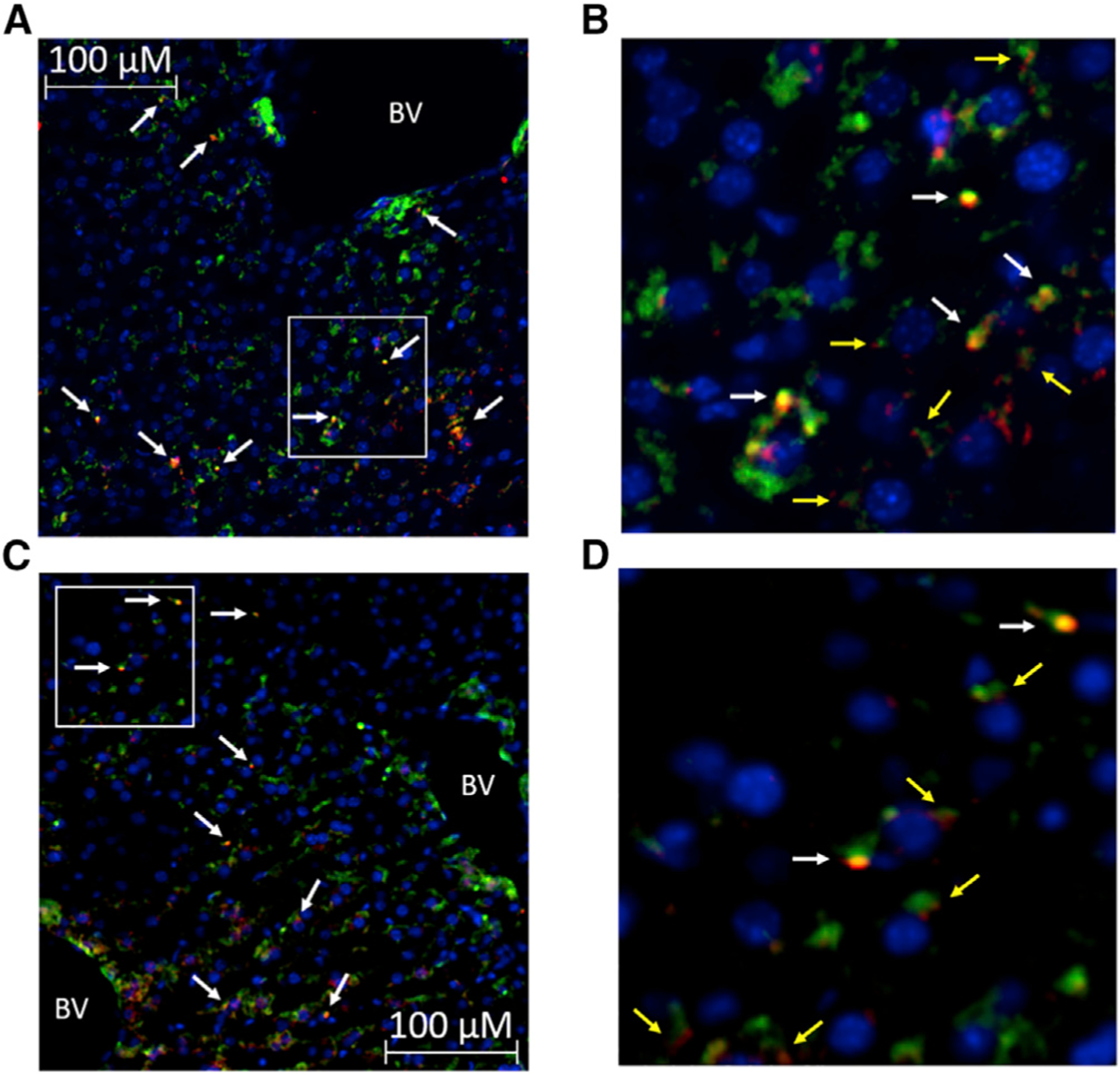
Immunohistochemical evidence of GABA_A_ receptor expressing vagal afferent innervation in the liver (A) Double labeling for the vagal afferent marker calretinin (green) and GABA_A_ receptors (red; arrows indicate co-staining). (B) Enlarged view of area within the white box in (A) (white arrows indicate co-staining, and yellow arrows indicate GABA_A_-positive staining immediately adjacent to calretinin-positive fibers). (C and D) Immunohistochemical staining for the alternative vagal afferent marker calcitonin gene-related peptide (CGRP, green) and GABA_A_ receptors (red, D; arrows indicate co-staining). Enlarged view of area within the white box in (C) (D; white arrows indicate co-staining, and yellow arrows indicate GABA_A-_positive staining immediately adjacent to CGRP-positive fibers). Blue represents DAPI (nucleus). Original magnification, 10×. BV, blood vessel.

Vagal afferent innervation in the liver has previously been identified using the vagal sensory immunohistochemical marker calretinin (Berthoud, 2004; Berthoud and Patterson, 1996). Calcitonin gene-related peptide (CGRP) has also been proposed as a marker of hepatic vagal afferent innervation, and hepatic CGRP staining is eliminated by capsaicin treatment (Tiegs et al., 1999) and substantially reduced following bilateral vagotomy (Sasaki et al., 1986). We confirmed the presence of both calretinin and CGRP-positive innervation in the mouse liver and established the presence of GABA_A_ receptors on calretinin and CGRP immunoreactive nerve fibers (Figures 6A–6D; Figure S5). Consistent with previous reports (Sasaki et al., 1986; Tiegs et al., 1999), the strongest degree of vagal afferent staining was evident in periportal areas while immunoreactive fibers penetrate into the liver parenchyma (Figures S5A and S5D). We then went on to confirm that liver calretinin and CGRP staining was decreased in hepatic vagotomized mice (Figure S6). We propose that elevated hepatic GABA release activates HVAN GABA_A_ receptors, causing chloride influx into vagal afferents and explaining the decrease in HVAN activity in response to hepatocyte depolarization (Figure 5).

### Associations between hepatic GABA system and glucoregulatory markers in humans with obesity

**Figure 7.**
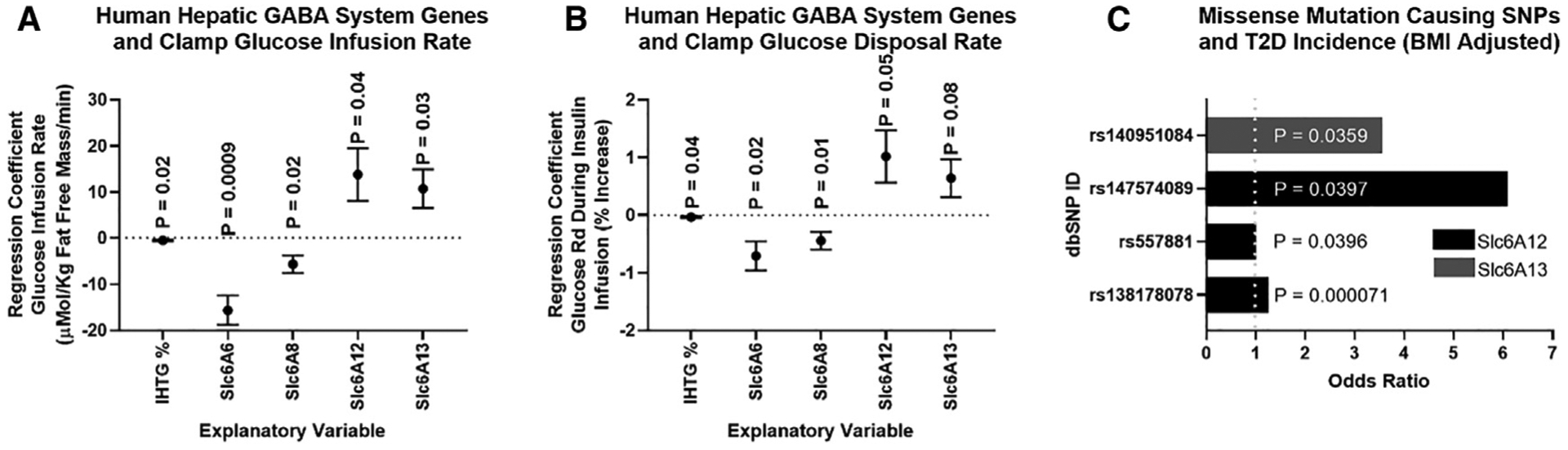
Associations between hepatic GABA system and glucoregulatory markers in obese humans (A and B) Multivariate regressions including intrahepatic triglyceride % (IHTG%) and the mRNA for the hepatic GABA transporters (Slc6A6, Slc6A8, Slc6A12, and Scl6A12) as explanatory variables for variations in glucose infusion rate during a hyperinsulinemic euglycemic clamp (μMol/kg fat-free mass/min) (A), and the glucose disposal rate calculated during a hyperinsulinemic-euglycemic clamp (Glucose Rd, % increase) (B). mRNA (fragments per kilobase of transcript per million mapped reads [FPKMs]) was quantified by RNA sequencing (RNA-seq) from liver tissue. (C) Single-nucleotide polymorphisms (SNPs) that cause missense mutations in Slc6A12 or Slc6A13 are associated with an increased incidence of type 2 diabetes (T2D) adjusted for body mass index (BMI). Regression data are presented as mean ± SEM.

To understand the potential clinical relevance of these findings, we assessed the hepatic mRNA expression of GABA transporters (*SLC6A6*, encodes for TauT; *SLC6A8*, encodes for the CRT; S*LC6A12*, encodes for BGT1; and S*LC6A13*, encodes for GAT2) in 19 people with obesity (age 45 ± 3 years, 2 men and 17 women) who were carefully characterized by measuring intrahepatic triglyceride (IHTG) content using magnetic resonance imaging (MRI) and insulin sensitivity using the hyperinsulinemic-euglycemic clamp procedure (HECP) in conjunction with stable isotopically labeled glucose tracer infusion. The subjects had a wide range in IHTG content, plasma insulin concentration, and measures of insulin sensitivity (Table S2). Our multivariate regression showed that IHTG (%) was negatively associated with both glucose infusion rate during a clamp (Figure 7A) and the percent increase in glucose rate of disposal from the basal state to the hyperinsulinemic clamp (Figure 7B; Table S3). Similarly, we found that hepatic S*LC*6A6 (Tau-T) and S*LC*6A8 (CRT) mRNA expression were negatively related to glucose infusion rate and insulin-induced percent increase in glucose disposal (Figures 7A and 7B). Finally, we observed that hepatic S*LC*6A12 and S*LC*6A13 expression were positively related to glucose infusion rate and insulin-stimulated enhancement of glucose disposal (Figures 7A and 7B). In turn, we hypothesize that BGT1 and GAT2 are primarily acting as GABA re-uptake transporters, which limit GABA signaling onto the HVAN, and that Tau-T and CRT are acting to export GABA, which encourages metabolic dysfunction. This hypothesized role of BGT1 and GAT2 in hepatic GABA re-uptake is supported by our explant data (Figure 4G). Of note, the summed expression of *SLC6A6* and *SLC6A8* was positively associated with IHTG %, suggesting that their expression increases with steatosis (p = 0.04).

We used the Accelerating Medicines Partnership Type 2 Diabetes Knowledge Portal to understand the effect of missense mutations in genes encoding the hepatic GABA transporters (*SLC6A6*, *SLC6A8*, *SLC6A12*, and *SLC6A13*). Missense mutations in the *SLC6A12* and *SLC6A13* genes are significantly associated with an increased incidence of type 2 diabetes (T2D) (Figure 7C; Table S4). Of note, a missense mutation inducing a pre-mature stop codon in the *SLC6A12* gene increases the odds ratio for T2D 15.8 times (dbSNP: rs199521597), establishing its key role in limiting the incidence of diabetes. Although, not specific to the liver, these genome-wide association study (GWAS) data do support the hypothesis that GABA transporters encoded by *SLC6A12* and *SLC6A13* act to prevent the development of T2D.

## DISCUSSION

We report neurotransmitter signaling by which hepatic steatosis may induce systemic insulin dysregulation, while establishing that hepatocyte GABA release is regulated by hepatocyte membrane potential. Using surgical and viral mouse models, we have established the key role of hepatic GABA production and release and hepatic vagal nerve signaling in the dysregulation of glucose homeostasis in obesity.

We propose a model that links hepatic lipid accumulation to HVAN activity and the development of hyperinsulinemia and insulin resistance (Figure 5). Hepatic lipid accumulation increases flux through gluconeogenesis and increases the hepatic FADH:FAD and NADH:NAD ratio (Figure 4H). The altered hepatic redox state inhibits the conversion of succinate to fumarate in the TCA cycle and instead drives succinate to SSA. SSA serves as substrate for GABA-T-mediated GABA production. Together with aspartate, the α-ketoglutarate formed by GABA-T produces oxaloacetate and glutamate, which feeds back into the GABA-T catalyzed reaction. The demand for gluconeogenic substrate and the high NADH:NAD ratio drives the carbons in oxaloacetate to malate and through gluconeogenesis. This gluconeogenic drive increases aspartate metabolism, explaining the decreased aspartate release in liver slices from obese mice (Figure 4J). Thus, gluconeogenic flux and a more reduced mitochondrial redox state direct the flow of intermediate molecules in obesity, resulting in elevated hepatic GABA production and increased aspartate utilization.

The ion dependence of GABA transport makes hepatocyte GABA export sensitive to changes in membrane potential. Because GABA transporters are sodium co-transporters, an increase in intracellular sodium ions and hepatocyte depolarization increases GABA export (Figure 4E). Obesity decreases hepatic ATP content (Figure 4D) and lowers Na^+^/K^+^-ATPase activity (Stanimirovic et al., 2018), providing a mechanism by which obesity depolarizes hepatocytes (Fitz and Scharschmidt, 1987) (Figure 2D) and encourages GABA export (Figure 4A). In fact, type 2 diabetics have lower hepatic ATP concentrations, and both peripheral and hepatic insulin sensitivity are significantly correlated with liver ATP concentrations (Schmid et al., 2011; Szendroedi et al., 2009). This model provides a rationale to explain why gluconeogenesis and hepatocyte depolarization are essential to the development of insulin resistance and hyperinsulinemia (Gómez-Valadés et al., 2008).

This research highlights the role of hepatocyte depolarization in diet-induced metabolic dysfunction. Under physiological conditions, hepatocyte membrane potential is closely regulated by insulin and glucagon. Acutely, insulin depolarizes while glucagon hyperpolarizes hepatocytes (Petersen, 1974; Wondergem, 1983). The hyperpolarizing effect of glucagon is proposed to be mediated by cAMP and is dependent on the Na^+^/K^+^ ATPase (Petersen, 1974). Accordingly, cAMP or glucagon counteracts insulin-stimulated hepatocyte depolarization (Hallbrucker et al., 1991; vom Dahl et al., 1991). HFD feeding decreases hepatic cAMP content in mice (Zingg et al., 2017). The decrease in hepatic cAMP, along with diminished ATP, may contribute to obesity-induced hepatocyte depolarization. Interestingly, a loss of hepatocyte membrane polarity has been implicated in the pathology of other disease states. Hepatocellular carcinoma is characterized by hepatocyte depolarization and increased GABAergic signaling, while increasing hepatocyte polarization protects against tumor proliferation (Sun et al., 2003). Thus, the regulation of hepatocyte membrane potential in healthy and disease states is critically tied to cellular and metabolic function.

Physiological concentrations of insulin and glucagon induce a 5- to 7-mV change in hepatocyte membrane potential (Petersen, 1974; Wondergem, 1983). This is comparable with the 6.86 ± 1.54 mV hyperpolarization induced by Kir2.1 expression (Figure 3B). Admittedly, the PSEM89S ligand maximally depolarized hepatocytes by 28 ± 5.4 mV (Figure 2E), which exceeds the depolarization observed in obesity (13 ± 4.7 mV; Figure 2D) and likely represents a supraphysiological response. However, at 10 min after PSEM89S ligand administration, when HVAN activity was first significantly depressed, hepatocyte depolarization was 17.0 ± 5.4 mV, representing a more physiological change in membrane potential.

Although the hypothesis that liver-derived signals communicate to the central nervous system via the HVAN is well supported in the literature (Bernal-Mizrachi et al., 2007; López-Soldado et al., 2017; Uno et al., 2012), the degree of hepatocyte vagal afferent innervation has remained controversial (Berthoud, 2004). Here, we provide evidence of vagal sensory innervation in close proximity to hepatocytes and have established the presence of GABA_A_ receptors on both calretinin and CGRP immunoreactive nerve fibers in the liver (Figures 6A–6D). Once exported, hepatic GABA can act at GABA_A_ receptors on vagal afferents to induce chloride influx and decrease firing rate (Yuan et al., 1998) (Figure 5), providing a connection between hepatic lipid accumulation and decreased HVAN activity.

We appreciate that it is counterintuitive that a decrease in HVAN activity causes hyperinsulinemia (Figures 2F, 2G, and 2J), yet surgical elimination of the HVAN limits obesity-induced hyperinsulinemia (Figure 1C). However, surgical vagotomy preserves activity of vagal afferent axons that terminate in the hindbrain. In fact, after vagotomy, NTS terminating axons show normal spontaneous activity, show normal signaling from the nodose ganglion to the NTS, and evoke normal postsynaptic excitatory currents in the NTS when electrically stimulated (Peters et al., 2013). Re-innervation of target tissues caudal to the severed vagus is minimal out to 45 weeks post-vagotomy (Phillips et al., 2003). Therefore, hepatic vagotomy eliminates the dysfunctional hepatocyte-vagal signaling in obesity, while preserving signaling from the HVAN above the surgical resection. Although hepatic vagotomy has been used extensively to investigate liver vagal denervation, several limitations must be acknowledged when interpreting this model (Berthoud et al., 1990; Berthoud and Neuhuber, 2000). We cannot exclude the possibility that the improvements in glucose homeostasis in response to hepatic vagotomy (Figure 1) are a result of interrupting non-vagal afferent signaling or partial loss of vagal efferent pancreatic innervation (Berthoud et al., 1990; Berthoud and Neuhuber, 2000).

Studies manipulating the hepatic vagal nerve propose that afferent parasympathetic signals from the liver may affect pancreatic insulin release. Activity of the HVAN is inversely related to parasympathetic efferent nerve activity at the pancreas, which stimulates insulin release (Lee and Miller, 1985; Nagase et al., 1993). Thus, portal glucose inhibits HVAN activity and increases pancreatic parasympathetic outflow to stimulate β cell muscarinic 3 receptor (M3R) signaling and insulin release (Duttaroy et al., 2004; Nagase et al., 1993). Accordingly, vagotomy reduces glucose-stimulated insulin secretion and basal hyperinsulinemia in obese rats by reducing cholinergic action on β cells (Balbo et al., 2016; Nagase et al., 1993). Furthermore, cholinergic blockade decreases basal serum insulin concentrations in obese, but not lean, mice, suggesting that elevated basal pancreatic parasympathetic efferent tone underlies obesity-induced hyperinsulinemia (Ahrén and Lundquist, 1982).

The HVAN also regulates insulin sensitivity (Spiridonov and Vorob’eva, 2000). Hepatic vagotomy acutely reduces insulin sensitivity in lean rats, decreasing skeletal muscle glucose clearance by 45% (Fernandes et al., 2011). In contrast, chronic hepatic vagotomy improves insulin sensitivity and glucose clearance in insulin-resistant mice (Bernal-Mizrachi et al., 2007) (Figure 1L). Furthermore, portal glucose delivery decreases HVAN firing activity and skeletal muscle glucose clearance (Galassetti et al., 1998; Niijima, 1982). We hypothesize that hepatic GABA production in obesity decreases HVAN activity to limit muscle glucose clearance and drive peripheral insulin resistance.

This work identifies enzymes involved in GABA production and transporters involved in hepatic GABA re-uptake and release as therapeutic targets for correcting the inherent metabolic disturbances in T2D.

## STAR★METHODS

### RESOURCE AVAILABILITY

#### Lead contact

Further information and requests for resources and reagents should be directed to and will be fulfilled by the lead contact, Benjamin Renquist (bjrenquist@arizona.edu).

#### Materials availability

The AAVs generated in this study can be ordered from Penn Vector Core.

#### Data and code availability

The datasets generate during this study are available at Mendeley Data: https://doi.org/10.17632/ck6hn5dpxy.1. The RNS-seq data have been deposited into the NCBI GEO database under accession number GEO: GSE144414.

### EXPERIMENTAL MODEL AND SUBJECT DETAILS

#### Animals

All studies excluding those done in albumin-cre expressing mice were conducted using male wild-type C57BL/6J purchased from Jackson Laboratories or bred in-house (Bar Harbor, ME). Albumin-cre male mice were purchased from Jackson Laboratories and crossed with wild-type females to generate in-house breeding of experimental albumin-cre expressing mice (Alb^Cre/+^) and sibling wild-type mice (Alb^+/+^). Mice were kept on a 14-hour light/10-hour dark schedule and housed 3–5 mice per cage until 1 week prior to study initiation, at which point animals were individually housed. We conducted studies in lean chow fed mice (7013 NIH-31, Teklad WI, 3.1 kcal/g, 18% kcal from fat, 59% kcal from carbohydrate, 23% kcal from protein) at 12–16 weeks of age. Studies in diet-induced obese sham and vagotomy mice were performed after 9 weeks on a high fat diet (TD 06414, Teklad WI, 5.1 kcal/g, 60.3% kcal from fat, 21.3% kcal from carbohydrate, 18.4% kcal from protein; 20–26 weeks of age). Studies in obese Kir2.1 expressing mice were performed at 3, 6, and 9 weeks after introduction of the high fat diet and all studies were repeated in 3 different cohorts. Kir2.1 and eGFP expressing mice weighing under 36 g after 9 weeks of high fat diet feeding were excluded from all data. Unless fasted, mice had *ad libitum* access to food and water. All studies were approved by the University of Arizona Institutional Animal Care and Use Committee.

#### Hepatic vagotomy surgeries

Surgeries were performed in 12-week old male C57BL/6J mice under isoflurane anesthesia. Mice were randomly assigned to a surgical group (sham or vagotomy). A ventral midline incision through the skin and peritoneum allowed us to isolate the hepatic vagus nerve as it branched from the esophagus (Figure 1A). In vagotomized mice, we severed the hepatic vagal nerve (Figure 1A, arrow A), while it remained intact in sham operated mice. The peritoneum was sutured with absorbable polyglactin 910 suture and the skin with nylon suture. Mice were given a single post-operative dose of slow release formulated buprenorphine analgesic (1.2 mg/kg slow release, sub-cutaneous). We monitored food intake and body weight daily and removed sutures 7 days post-operation.

#### Viral induced channel expression

The depolarizing channel (PSAM^L141F,Y115F^-5HT3HC), originally engineered by Dr. Scott Sternsons group (Magnus et al., 2011), was made by mutating the acetylcholine binding domain of a chimeric channel that included the binding domain of the α7 nicotinic acetylcholine receptor and the ion pore domain of the serotonin receptor 3a. The ligand binding domain mutations (Leu^141^ → Phe and Tyr^115^ → Phe) limited the agonist action of acetylcholine and allowed for stimulation by a pharmacologically selective effector molecule PSEM89S. The exogenous ligand PSEM89S opens the serotonin receptor 3a channel allowing Na^+^, K^+^, and Ca^2+^ passage into the cell and membrane depolarization. AAV8 viral vectors were used for plasmid delivery in all the reported studies and were synthesized by the Penn Vector Core. Hepatic specific expression of the depolarizing channel was achieved through two different methods. First, expression of a cre-recombinase dependent depolarizing channel was driven by a globally expressed CAG promoter. LoxP sites limited expression to cre-recombinase expressing tissue, and tail vein injection of 1×10^10^ viral genome copies established hepatocyte expression in albumin-cre but not wild-type mice. Second, a separate AAV8 viral vector, induced hepatic specific expression of the same depolarizing channel by driving expression using the thyroxine binding globulin (TBG) promoter. Tail vein injection of 1×10^11^ viral genome copies established hepatocyte expression (Figure 2C; Figure S1B). The thyroxine binding globulin promoter also drove hepatic expression of the hyperpolarizing, inward-rectifier K^+^ channel, Kir2.1. Tail vein injection of 1×10^11^ viral genome copies established hepatocyte specific expression (Figure 3A). To confirm channel expression and tissue specificity, all viral vector plasmids encoded for enhanced green fluorescent protein (eGFP).

#### Human subjects

A total of 19 men and women with obesity (age 45 ± 3 years old, 2 men and 17 women) who were scheduled for bariatric surgery at Barnes-Jewish Hospital in St. Louis, MO participated in this study, which was conducted at Washington University School of Medicine in St. Louis. Subjects provided written, informed consent before participating in this study, which was approved by the Human Research Protection Office at Washington University School of Medicine in St. Louis, MO (ClinicalTrials.gov: NCT00981500).

### METHOD DETAILS

#### PSEM89S ligand injection studies

All studies in virus injected mice were conducted at least 5 days post virus injection to allow for maximal channel expression. Individually housed mice were intraperitoneally injected with the ligand for the depolarizing channel (PSEM89S; 30mg/kg; 0.1mL/10 g body weight) or PBS (0.1mL/10 g body weight).

Studies conducted in mice injected with the cre-dependent depolarizing channel virus took place at 8 am. Food was removed upon study initiation. Blood for serum insulin and glucose determination was collected from the tail vein 15 minutes following intraperitoneal injection. All mice received both saline and PSEM89S ligand injection on separate days.

Studies conducted in mice expressing the cre-independent depolarizing channel began at 1 pm following a 4 hour fast. Mice received an oral glucose gavage (2.5 g/kg) 10 minutes after intraperitoneal injection of the PSEM89S ligand or saline. 15 minutes following glucose administration (25 minutes post treatment injection), blood for serum insulin and glucose determination was collected from the tail vein. All mice received both saline and PSEM89S ligand injection on separate days. These studies were repeated in 2 cohorts.

#### Electrophysiology

We performed simultaneous *in vivo* recordings of hepatocyte membrane potential and hepatic afferent vagal nerve firing activity in anesthetized (isoflurane) mice to directly assess the effect of hepatocyte depolarization on hepatic afferent vagal nerve activity. The abdomen was shaved and scrubbed with betadine and isopropanol before an incision through the skin and peritoneum was made to expose the internal organs. The intestines were moved to expose the liver and one lobe of the liver was secured onto a small platform to minimize movement caused by respiration. A ground electrode was secured under the skin and the hepatic vagal nerve was gently lifted onto a hook-shaped electrode (Figure 1A, arrow A) attached to the positive pole of a Grass P511 AC coupled amplifier, and the signal was filtered with a bandwidth of 300–1000 Hz. The nerve and hook electrode were dried and surrounded with ice cold kwik-sil to secure placement of the hook. The hepatic vagal nerve to the right of the hook, near the esophagus was cut to eliminate efferent firing (Figure 1A, arrow B). Once the kwik-sil had set, the anesthetized mouse was bathed in 37°C Krebs-Henseleit (KH) buffer gassed with CO2. After placement and sealing of the hook electrode, 45–60 minutes of basal nerve activity was monitored/recorded with pClamp software (version 10.2; Molecular Devices) until nerve activity stabilized, after which, we began treatments.

Simultaneously, intracellular recordings of hepatocytes were made with sharp glass electrodes (30–40 MΩ) pulled from thin-walled borosilicate glass capillary tubes (OD: 1 mm; ID: 0.78mm; Sutter Instrument Co., Novato, CA), filled with 1.5M KCl and positioned visually using a motorized 4-axis micromanipulator (Siskiyou, Grants Pass, OR). Electrical signals were conducted via an Ag–AgCl electrode connected to a headstage (Axoclamp ME-1 probe), which was in turn connected to an Axoclamp 2B amplifier. Both nerve and intracellular signals were sent to an A/D converter (Digidata 1322A, Molecular Devices, Sunnyvale, CA), digitized at 20 kHz and viewed on a computer monitor using pClamp software (version 10.2; Molecular Devices).

Before treatments were applied hepatocyte impalement was determined by an abrupt negative deflection upon penetration of the cell and a stable intracellular potential (−45 to −25 mV for mouse hepatocytes) for at least 2 minutes. If the recording of hepatocyte membrane potential was not stable the electrode was removed and membrane potential was measured on another hepatocyte.

To assess the response to channel activation, PSEM89S ligand was bath applied (30 μM) for 45 min during recordings. In order to understand the effect of Kir2.1 channel on hepatocyte membrane potential, a 10-minute baseline measure was collected and then Barium (BaCl 50 μM) was bath applied and recording continued for 45 minutes. Barium blocks Kir2.1 mediated current, thus the change in membrane potential in response to barium indicates the degree of hyperpolarization resulting from Kir2.1 channel expression. In all electrophysiology studies mice were sacrificed by cutting the diaphragm and subsequent cervical dislocation. Tissues were collected to confirm tissue specificity of channel expression and the % of hepatocytes that were expressing the channel. All studies were performed at room temperature (25°C).

To assess the response to GABAA receptor activation, we again used the isoflurane anesthetized mouse and similarly placed the hepatic vagal nerve on a hook electrode that was electrically isolated by kwik-sil. We used warmed saline to keep the organs moist. Potential efferent signals were eliminated by severing the nerve near the esophagus. After 5 minutes of baseline recording, we delivered 1 mL of 100 μM muscimol and continuously measured vagal nerve activity for 6 minutes, we then delivered 1 mL of 2 mM muscimol and continued vagal nerve recording for 13 minutes. Mice were sacrificed by cervical dislocation at the conclusion of recording. For these studies vagal nerve activity was assessed using LabChart software (AD Instruments, Colorado Springs, CO, USA). Filters were applied to eliminate data artifacts resulting from breathing and cardiovascular signals and the rectified integrated signal was calculated per unit of time recorded (Figure S8).

#### Immunohistochemistry and imaging

To confirm the specificity and extent of viral-induced channel expression in hepatocytes, immunohistochemistry for GFP was performed. Liver, adipose, pancreas, and skeletal muscle were collected into 4% paraformaldehyde in 0.1 M PBS (Phosphate Buffered Saline) immediately after sacrifice. After 4 h at 4°C, tissues were transferred to a 30% sucrose solution in 0.1 M PBS and kept at 4°C until tissues sunk to the bottom of the solution. Tissues were snap frozen on liquid nitrogen in OTC (Optimal Cutting Temperature; Sakura Finetek USA Inc, Torrance, CA) and stored at −80°C. We used a cryostat HM 520 (MICROM International GmbH, Walldorf, Germany) to get 10 μM thick slices which we collected onto Superfrost Plus slides. Immunohistochemistry for GFP alone (Figures 2A–2C; Figures S1A and S1B) was performed as follows: Briefly, slides were washed twice in PBS and twice in PBST (3% Triton in PBS) before being exposed to blocking solution (5% normal goat serum in PBST) for 1 h. Slides were subsequently exposed to a 1:5000 dilution of the primary anti-GFP antibody in blocking solution (Alexa488-conjugated rabbit anti-GFP; Life Technologies, Waltham, MA) for 3 hours at room temperature. After primary antibody incubation we washed the slides 3 times in PBST and 2 times in PBS prior to placing the coverslip with DAPI Fluoromount-G as the mounting medium (SouthernBiotech, Birmingham, AL). Fluorescent imaging was performed without antibody amplification in mice administered the AAV8 that encoded for Kir2.1 and tdTomato. Immunohistochemistry for GFP and the hepatocyte specific marker arginase-1 (Figures S1C–S1E) was performed as follows: Slides were washed twice in PBS and twice in PBST (3% Triton in PBS) before being exposed to blocking solution (5% normal donkey serum in PBST) for 1 h. Slides were then incubated overnight at 4°C in a 1:400 dilution of the primary anti-arginase-1 antibody in blocking solution (Rabbit anti-liver arginase ab91279; Abcam, Cambridge, UK). After overnight primary antibody incubation slides were washed 5 times with PBST and exposed to a 1:500 dilution of the primary anti-GFP antibody (Alexa488 conjugated mouse anti-GFP sc-9996 AF488; Santa Cruz, Dallas, TX) and the secondary anti-rabbit antibody (Alexa568 conjugated donkey anti-rabbit A10042; Thermo Fisher, Waltham, MA) in blocking solution and for 1 hour at room temperature. Slides were then washed 5 times in PBST and 2 times in PBS prior to applying DAPI and a coverslip. Immunohistochemistry for calcitonin gene-related peptide (CGRP) and GABA_A,_ and calretinin and GABA_A_ were performed identical to that described for GFP and arginase-1 with the following primary anti-CGRP and anti-GABA_A_ antibodies at a 1:100 dilution (Goat anti-CGRP ab36001 and rabbit anti-GABA_A_ α5 ab10098; Abcam, Cambridge, UK) and the primary anti-calretinin antibody at a final concentration of 15 μg/mL (Goat anti-calretinin AF5065; R&D Systems, Inc., Minneapolis, MN). The secondary anti-rabbit and anti-goat antibodies were used at a 1:500 dilution (Alexa568 conjugated donkey anti-rabbit A10042 and alexa488 conjugated donkey anti-goat A32814; Thermo Fisher, Waltham, MA). Images were collected by fluorescent microscopy (Leica DM5500B, Leica Microsystems, Wetzlar, Germany), captured using HCImage Live, and formatted in Image-Pro Premier 9.2. 10X magnification was used to ensure a wide field of vision and accurate assessment of degree of expression. 20X magnification was used to image co-staining for GFP and arginase-1.

#### Body composition

Body Composition was measured at 0 and 9 weeks after high fat diet exposure in non-anesthetized mice using the EchoMRI^™^ 1100 (EchoMRI, LLC, Houston, TX, USA).

#### Glucose tolerance test

Oral glucose (2.5g/kg; 0.1mL/10 g body weight; Chem-Impex Int’l Inc., Wood Dale, IL) was given to 4 hour fasted individually housed mice. All glucose tolerance tests began at 1 pm and glucose was measured in whole blood, collected from the tail vein, by glucometer (Manufacture # D2ASCCONKIT, Bayer, Leverkusen, Germany) at 0, 15, 30, 60, 90, and 120 minutes after glucose gavage. Blood for serum insulin (oral glucose stimulated insulin secretion; OGSIS) and glucose determination was collected from the tail vein 15 minutes following glucose administration.

#### Insulin tolerance test

Intraperitoneal insulin (0.75U/kg; 0.1mL/10 g body weight; Sigma Aldrich, St. Louis, MO) was given to 4 hour fasted individually housed mice. All insulin tolerance tests began at 1 pm and glucose was measured in whole blood, collected from the tail vein, by glucometer (Manufacture # D2ASCCONKIT, Bayer, Leverkusen, Germany) at 0, 30, 60, 90, and 120 minutes after insulin injection.

#### Pyruvate tolerance test

Intraperitoneal sodium pyruvate (1.5g/kg; 0.1mL/10 g body weight; Alfa Aesar, Ward Hill, MA) was given to 16 hour fasted individually housed mice. Mice were switched to wood chip bedding (Harlan Laboratories; Cat. # 7090 Sani-Chips) at the initiation of the fast. All pyruvate tolerance tests began at 9 am and the rise in glucose was measured in whole blood, collected from the tail vein, by glucometer (Manufacture # D2ASCCONKIT, Bayer, Leverkusen, Germany) at 0, 30, 60, 90, and 120 minutes after pyruvate injection. This is indicative of hepatic gluconeogenic potential from pyruvate.

#### Serum assays

Within 2 hours of collection, blood was left to clot at room temperature for 20 minutes. Thereafter the blood was centrifuged at 3,000xg for 30 minutes at 4°C and serum was collected. Serum was stored at −80°C until metabolite and hormone analyses. Serum glucose was analyzed by colorimetric assay (Cat. # G7519, Pointe Scientific Inc., Canton MI). Serum insulin was analyzed by enzyme-linked immunosorbent assay (ELISA; Cat. # 80-INSMSU-E01,E10, Alpco, Salem, NH). Serum glucagon was analyzed by enzyme-linked immunosorbent assay (ELISA; Cat. # 10-1281-01, Mercodia, Uppsala, Sweden) from tail vein blood collected at 9 am from fed mice (Vagotomy study) or after a 4 hour fast at 1 PM (Kir2.1 study).

#### Liver slice explant studies

Liver slices from experimental mice were incubated *ex vivo* to measure release of signaling molecules. A peristaltic pump perfusion system was used to deliver warmed KH buffer to the liver through the portal vein. Briefly, mice were anesthetized with an intraperitoneal injection of ketamine (10mg/mL) and diazepam (0.5mg/mL). Once mice were unresponsive, an incision in the lower abdomen through the skin and peritoneal membrane was made vertically through the chest along with transverse incisions on both sides to expose the liver. A 30-gauge needle was inserted into the hepatoportal vein to blanch the liver. The inferior vena cava was cut to relieve pressure in the circulatory system and allow blood to drain. The perfusion continued for several minutes at a rate of 4mL/minute until the liver was completely blanched. The liver was removed and washed in warm PBS before being sliced into 0.2 mm slices using a Thomas Sadie-Riggs Tissue Slicer. Two liver slices were taken from each mouse. Tissue slices were placed individually into a well on a 12-well plate pre-filled with 1mL of KH buffer that had been sitting in an incubator set to 37°C and gassed with 5% CO_2_. Liver slices were incubated in the initial well for 1 hour to stabilize before being transferred to a fresh well pre-filled with KH buffer. Liver slices treated with the GABA-T inhibitor EOS were incubated in media containing EOS (5.3 mM) during the second hour of incubation. Liver slices treated with the Na^+^/K^+^ ATPase inhibitor ouabain were incubated in media containing ouabain (1 mM) media was collected after 15 minutes of incubation. Liver slices treated with the GABA transporter inhibitors for BGT1 and GAT2 were incubated in media containing betaine (1 mM) or Nipecotic acid (1 mM) or both during the second hour of incubation. Liver slices incubated in reduced and low NaCl media sat in normal KH buffer (NaCl 118 mM) for the first hour and then were transferred to reduced (60 mM) or low NaCl (15 mM) KH buffers for the second hour. For the reduced and low NaCl medias, respectively, 58 and 103 mM of NaCl were replaced with 116 and 206 mM of mannitol to maintain the osmolarity of the buffer. After 1 hour in the second well, tissue and media were collected. Liver slice samples and KH media samples from both wells of each mouse were pooled. Liver slices were snap frozen in liquid nitrogen, while media was frozen and stored at −80°C for future analysis.

#### Explant media analysis

Preliminary media samples were sent to the Mayo Clinic Metabolomics Regional Core for mass spectrophotometry analysis using their neuromodulators panel (Table S1). For all liver slice GABA and aspartate release data, we thawed the media collected from the *ex vivo* hepatic slice culture on ice and centrifuged for 5 minutes at 10,000xg at 4°C to remove tissue debris. We then measured GABA in the supernatant using a commercially available ELISA (REF# BA E-2500, Labor Diagnostika Nord, Nordhorn, Germany).

Aspartate release was measured using liquid chromatography-mass spectrometry. Samples were prepared for analysis by LC-MS/MS using protein precipitation. Twenty μl of each sample and standard curve increment was transferred to 1.5 mL tubes. One hundred eighty μl acetonitrile (ACN) was added to each tube followed by a 5 s vortex. All samples were incubated at 4°C for one hour for precipitation. Samples were then centrifuged at 10,000 RPM for 10 minutes and the supernatant transferred to 300 μL HPLC vials for analysis. The aqueous portion of the mobile phase was buffered using 10 mM ammonium bi-carbonate with the pH adjusted to 7.4 using 1M formic acid and ammonium hydroxide. Methanol was used as the organic portion of the mobile phase. The column for separation was a Phenomenex Luna Silica (2) with 5 μm particle diameter and 100Å pore size. Column internal diameter was 4.6 mm and length was 150 mm. A Shimadzu LC10 series HPLC with two dual piston pumps was used for sample injection and solvent delivery. The flow rate was fixed at 300 μL per minute. Aspartate was quantified using an LTQ Velos Pro mass spectrometer. Eluate from the Shimadzu HPLC was ionized using a Thermo ESI source. Source voltage was 6 kV; sheath and auxiliary gas flows were 40 and 20 units respectively. The ion transfer capillary was heated to 300°C. The LTQ Velos Pro was operated in negative SRM mode using two transitions: 132.1- > 115 for quantification and 132.1- > 88.1 as a qualifier. Data integration and quantification were performed using the Thermo Xcalibur software packaged with the LTQ Velos Pro.

#### Liver analyses

Prior to analysis, frozen livers were powdered using a liquid nitrogen cooled mortar and pestle to obtain homogeneous liver samples. To measure liver DNA content (ng dsDNA/g tissue), 10–20 mg of powdered liver was sonicated in 200 μL DEPC H_2_O and dsDNA determined by fluorometric assay (Cat. # P7589, Invitrogen, Waltham, MA).

Total liver lipids were extracted from powdered liver samples. Briefly, 10–20 mg of sample was sonicated in 100μL PBS. 1 mL of 100% ethanol was added to each sample and vortexed for 10 minutes. Following 5 minutes of centrifugation at 16,000xg at 4°C, supernatant was transferred to a fresh tube for analysis of liver triglycerides (Cat. # T7531, Pointe Scientific Inc., Canton, MI).

Hepatic NADH and NAD were quantified by fluorometric assay (ab176723, Abcam, Cambridge, UK). Hepatic ATP concentrations were assessed as previously described (Geisler et al., 2016).

#### Studies conducted in human subjects

Intrahepatic triglyceride content was determined by using magnetic resonance imaging (3.0-T superconducting magnet; Siemens, Iselin, NJ) in the Center for Clinical Imaging Research. A 7-hour (3.5-h basal period and 3.5-h insulin infusion period) hyperinsulinemic-euglycemic clamp procedure (HECP), in conjunction with stable isotopically labeled glucose tracer infusion, was then conducted in the Clinical Translational Research Unit (CTRU), as previously described (Korenblat et al., 2008). This procedure was performed to determine whole-body insulin sensitivity, which was assessed as the glucose infusion rate (expressed as mg·kg FFM^−1^·min^−1^) during during the last 30 minutes of the HECP; and skeletal muscle insulin sensitivity, which was assessed as the percent increase in the rate of glucose disposal above basal, during the last 30 minutes of the HECP (Korenblat et al., 2008). For liver RNA sequencing (RNA-seq), liver tissue was obtained by needle biopsy during the bariatric surgical procedure, before any intra-operative procedures were performed. Liver tissue was rinsed in sterile saline, immediately frozen in liquid nitrogen, then stored at −80°C until RNA extraction. Total RNA was isolated from frozen hepatic tissue samples by using Trizol reagent (Invitrogen, Carlsbad, CA). Library preparation was performed with total RNA and cDNA fragments were sequenced on an Illumina HiSeq-4000. The fragments per kilobase million reads upper quartile (FPKM-UQ) values were calculated and used for further gene expression analyses. All RNA-seq data used in this study have been deposited into the NCBI GEO database under accession number GSE144414.

### QUANTIFICATION AND STATISTICAL ANALYSIS

#### Statistics

We analyzed the data in SAS Enterprise Guide 7.1 (SAS Inst., Cary, NC), using a mixed model ANOVA for all analyses. ANOVA tests do not have a one-tailed versus two-tailed option, because the distributions they are based on have only one tail. When comparisons between all means were required, we used a Tukey’s adjustment for multiple comparisons. When comparisons of means were limited (e.g., only within a time point or treatment), we used a bonferonni correction for multiple comparisons. For the analysis of ITT, OGTT, and PTT repeated-measures ANOVA were performed by including time point in the analysis. When applicable analyses were conducted separately for chow and HFD fed mice. In the Kir2.1 mice, which were monitored for response at 0, 3, 6, and 9 weeks, analyses were performed for each time point individually. For analysis of cre-dependent depolarizing channel effects, analysis was performed in each genotype separately (Alb^cre/+^ or Alb^+/+^) and the main effect was injection (PSEM89S ligand or saline). For analysis of the studies using the thyroxine binding promoter driven ligand gated depolarizing channel we had the main effect of injection (PSEM89S ligand or saline). For the vagotomy analyses the main effect was surgery (sham or vagotomy) and weeks on high fat diet when applicable. For the Kir2.1 analyses the main effect was virus (eGFP or Kir2.1). Linear regressions of body weight and serum insulin concentrations were performed on Kir2.1 and eGFP controls, and sham and vagotomized mice using SAS Enterprise Guide 7.1. All insulin tolerance tests are presented as a percentage of baseline glucose in main and supplemental figures, and additionally presented as raw glucose values in Figure S7. Human data was analyzed using a multivariate regression including IHTG content, and *SLC6A6, SLC6A8, SLC6A12*, and *SLC6A13* mRNA expression as independent variables with Type 3 test of fixed effects used to determine significance and estimates derived from maximum likelihood estimation. All graphs were generated using GraphPad Prism 8 (GraphPad Software Inc., La Jolla, CA). Information on replicates and significance is included in the figures. Error bars are defined in the figure legends.

## Supplementary Material

1

## Figures and Tables

**Table:** KEY RESOURCES TABLE

REAGENT or RESOURCE	SOURCE	IDENTIFIER
Antibodies		
Alexa488 conjugated rabbit anti-GFP	Life Technologies	Cat # A-21311, RRID:AB_221477
Rabbit anti-liver arginase	Abcam	Cat # ab91279, RRID:AB_10674215
Alexa568 conjugated donkey anti-rabbit	Thermo Fisher	Cat # A10042, RRID:AB_2534017
Goat anti-CGRP	Abcam	Cat # ab36001, RRID:AB_725807
Rabbit anti-GABA_A_ α5	Abcam	Cat # ab10098, RRID:AB_296840
Goat anti-calretinin	R&D Systems	Cat # AF5065, RRID:AB_2068516
Alexa488 conjugated donkey anti-goat	Thermo Fisher	Cat # A32814, RRID:AB_2762838
Bacterial and virus strains		
TBG driven PSEM89S ligand depolarizing channel, cre-independent virus	Penn Vector Core	AAV8.TBG PI.PSAM(Y115F,L141F)a75HT3-HC.IRES.GFP.WPRE.bGH (RRID: Addgene_32477)
Kir2.1 Virus	Penn Vector Core	AAV8.TBG.PI.Kir2.1-T2AtdTomato.WPRE.bGH (Addgene_60661)
eGFP Control Virus	Penn Vector Core	AAV8.TBG.PI.eGFP.WPRE.bGH
Cre-dependent PSEM89S ligand depolarizing channel virus	Penn Vector Core	AAV8.CAG.Flex.PSAM(Y115F,L141F)a75HT3-HC.IRES.GFP.SV40
Chemicals, peptides, and recombinant proteins		
D-glucose	Chem-Impex Int’l Inc.	Cat # 00805
Insulin	Sigma Aldrich	Cat # 10516-5ML
Sodium Pyruvate	Alfa Aesar	Cat # A11148-18
Ouabain	Sigma Aldrich	Cat # O3125 – 250 MG
Betaine hydrochloride	Sigma Aldrich	Cat # B3501
Nipecotic Acid	Alfa Aesar	Cat # B24723
Ethanolamine-O-sulfate (EOS)	Sigma Aldrich	Cat # 06720-100G
DAPI Fluoromount-G®	Southern Biotech	Cat # 0100-20
PSEM89S	Scott Sternson	N/A
Critical commercial assays		
Glucose Oxidase Assay	Pointe Scientific Inc.	Cat # G7519
Insulin ELISA	Alpco	Cat # 80-INSMSU-E01,E10
GABA ELISA	Labor Diagnostika Nord	Cat # BA E-2500
Glucagon ELISA	Mercodia	Cat # 10-1281-01
Quant-iT PicoGreen dsDNA Assay Kit	Invitrogen	Cat # P7589
Triglyceride Assay	Pointe Scientific Inc.	Cat # T7531
NAD/NADH Assay	Abcam	ab176723
Deposited data		
Raw Data	This Paper	Mendeley Data: https://doi.org/10.17632/ck6hn5dpxy.1
RNA seq Data	This Paper	NCBI GEO database accession number GEO: GSE144414
Experimental models: Organisms/strains		
Mouse: C57BL6/J	Jackson Laboratories	JAX stock 000664
Mouse: B6. Cg-Speer6-ps1^Tg(Alb-cre)21Mgn^/J	Jackson Laboratories	JAX stock 003574
Software and algorithms		
SAS Enterprise Guide 7.1	SAS Institute Inc.	https://www.sas.com/en_us/home.html
GraphPad Prism 8	GraphPad Software Inc.	https://www.graphpad.com/
Other		
Mouse Chow Food	Teklad	7013 NIH-31
Mouse 60% High Fat Diet Food	Teklad	TD 06414

## Highlights

- The hepatic vagal nerve affects glucose clearance and insulin sensitivity
- Hepatocyte membrane potential regulates serum insulin and insulin sensitivity
- GABA transport into and out of the hepatocyte relies on electrogenic transporters
- Hepatic vagal nerve activity is inhibited by GABA_A_ receptor stimulation
