# Supplementary material for: Hepatocyte membrane potential regulates serum insulin and insulin sensitivity by altering hepatic GABA release
Source: Cell Rep. Author manuscript; Available in PMC 2021 Aug 5. (PMC8341405; doi:10.1016/j.celrep.2021.109298)
Supplement: 1 [file NIHMS1728619-supplement-1.pdf]

Cell Reports, Volume *35*

Supplemental information

Hepatocyte membrane potential regulates serum insulin and insulin sensitivity by altering hepatic GABA release

Caroline E. Geisler, Susma Ghimire, Chelsea Hepler, Kendra E. Miller, Stephanie M. Bruggink, Kyle P. Kentch, Mark R. Higgins, Christopher T. Banek, Jun Yoshino, Samuel Klein, and Benjamin J. Renquist

Supplemental Data Titles and Legends

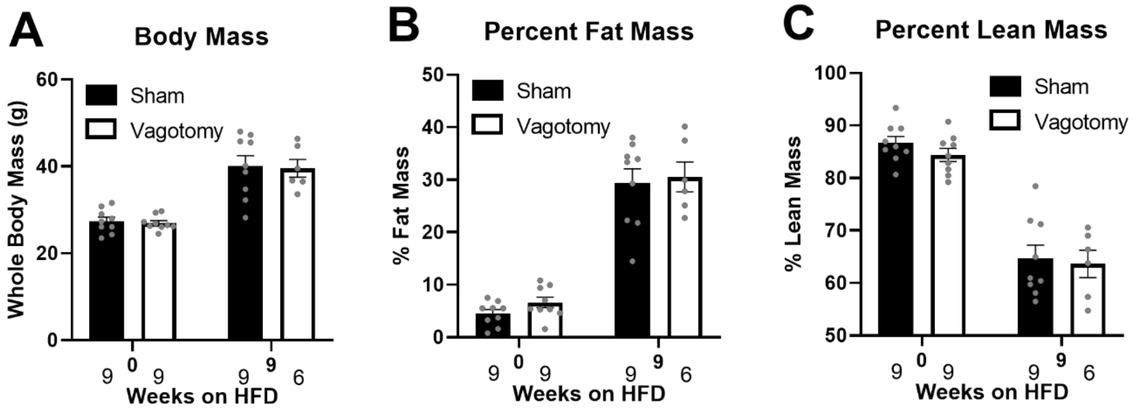

Figure S1. Related to Figure 1. Hepatic vagotomy did not affect body mass (A), percent fat mass (B), or percent lean mass (C) in adult male mice on a chow diet and after 9 weeks on a high fat diet. Number below bar denotes n per group. All data are presented as mean ± SEM.

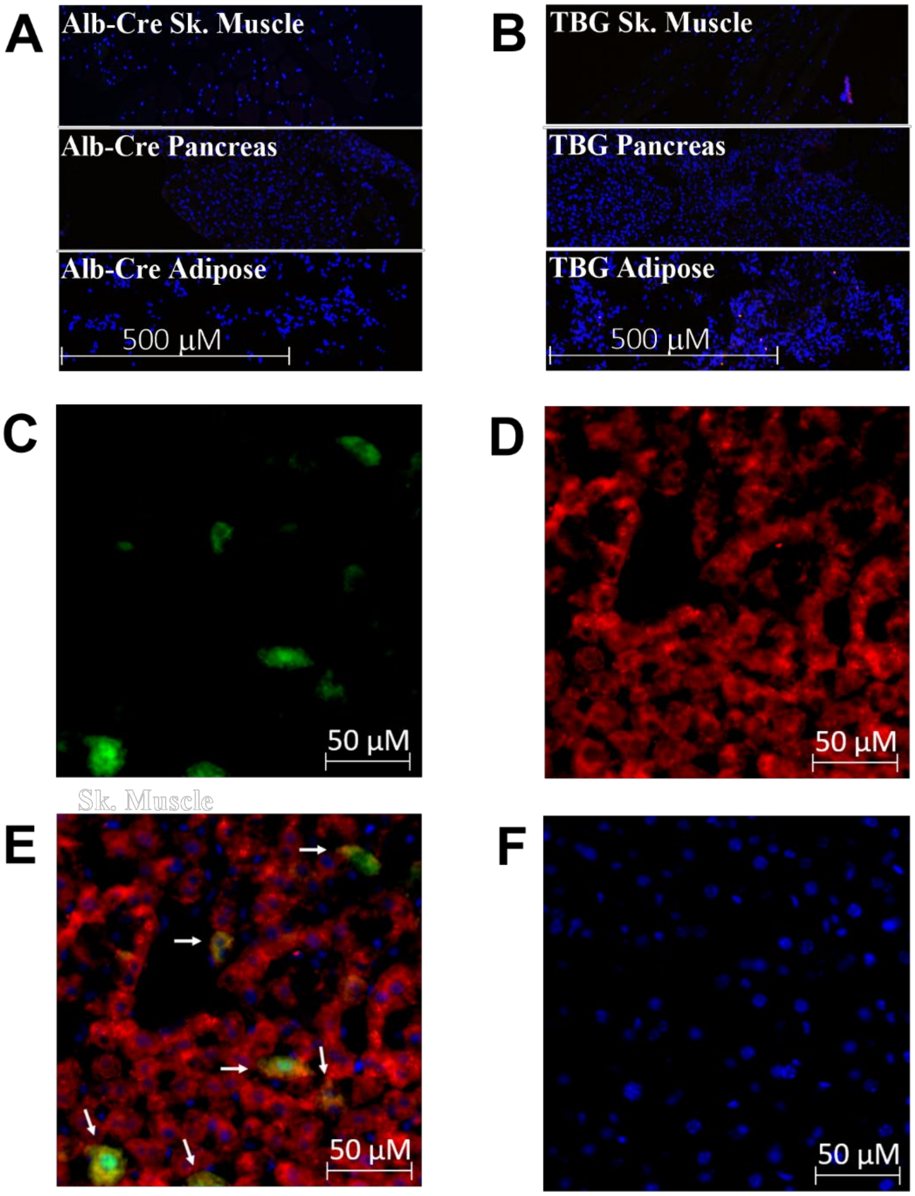

Figure S2. Related to Figure 2. Immunohistochemical validation of liver specific viral induced PSEM89S ligand gated depolarizing channel (A-B; 10X magnification). Skeletal muscle (Sk. Muscle), pancreas, and adipose tissue from an albumin-cre expressing mouse tail-vein injected with an AAV8 encoding for the PSEM89S ligand activated depolarizing channel and green fluorescent protein (GFP) whose expression is dependent on cre-recombinase (A). Skeletal muscle (Sk. Muscle), pancreas, and adipose from a wildtype mouse tail-vein injected with an AAV8 encoding the PSEM89S ligand activated depolarizing channel and GFP whose liver specific expression is driven by the thyroxine binding globulin (TBG) promoter (B). GFP positive cells in the liver of a wildtype mouse tail-vein injected with the TBG virus co-stain with arginase-1 (C-E; 20X magnification). Staining for GFP (C), the hepatocyte specific marker arginase-1 (D), and double labeling of GFP and arginase-1 (E; arrows indicate co-staining). No primary control imaged at the same settings as panel E (F). Green = GFP, red = arginase-1, blue = DAPI (nucleus).

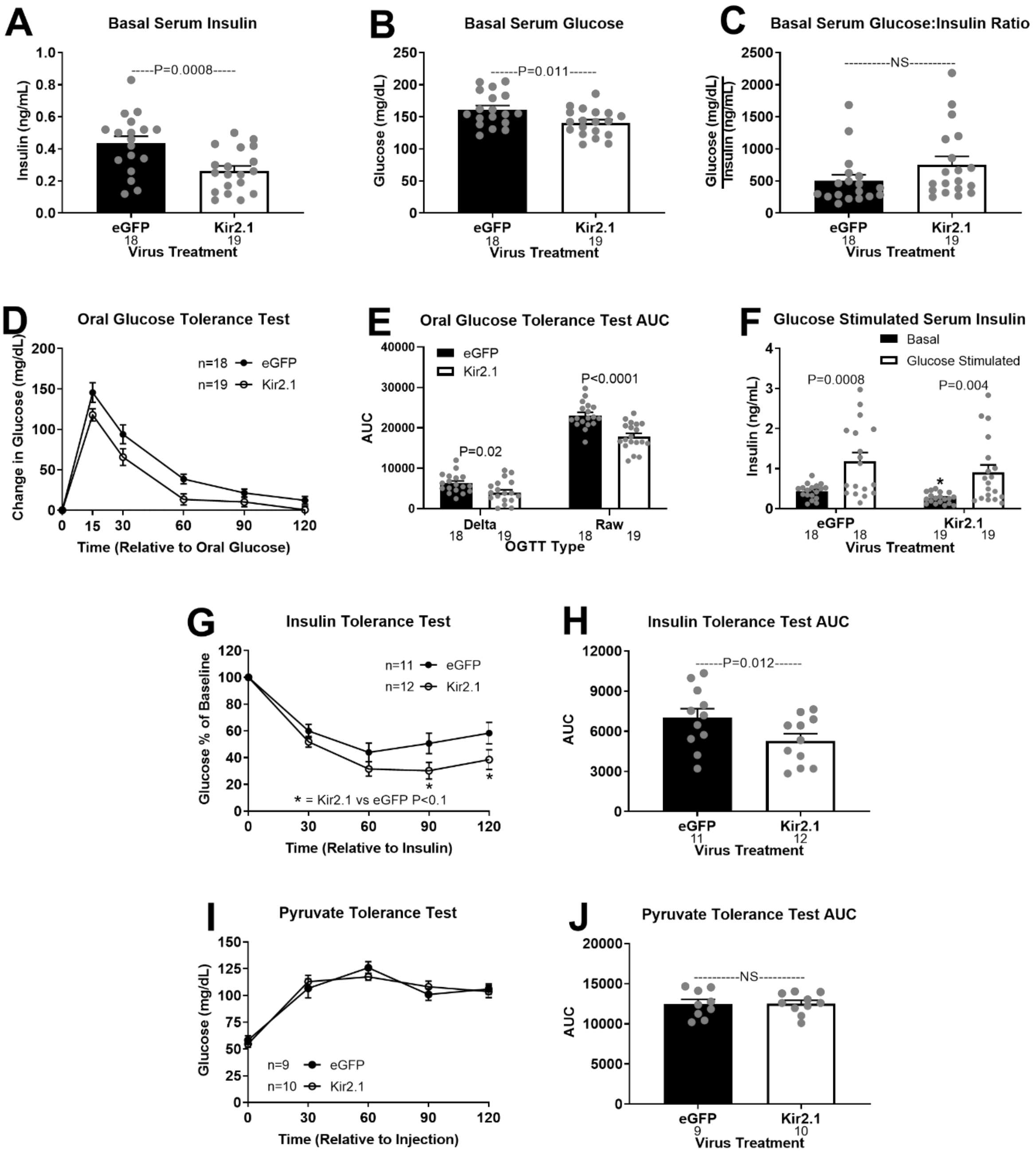

Figure S3. Related to Figure 3. Hepatic Kir2.1 expression alters glucose homeostasis in the lean mouse. Hepatic Kir2.1 expression effects on serum insulin (A) glucose (B), glucose:insulin ratio (C), oral glucose tolerance (OGTT; D), OGTT area under the curve (AUC; E), oral glucose stimulated serum insulin (F; * denotes significance ($P < 0.05$) between bars of the same color), insulin tolerance (ITT; G) ITT AUC (H), pyruvate tolerance (PTT; I), and PTT AUC (J). NS = non-significant. Number below bar denotes n per group. All data are presented as mean ± SEM.

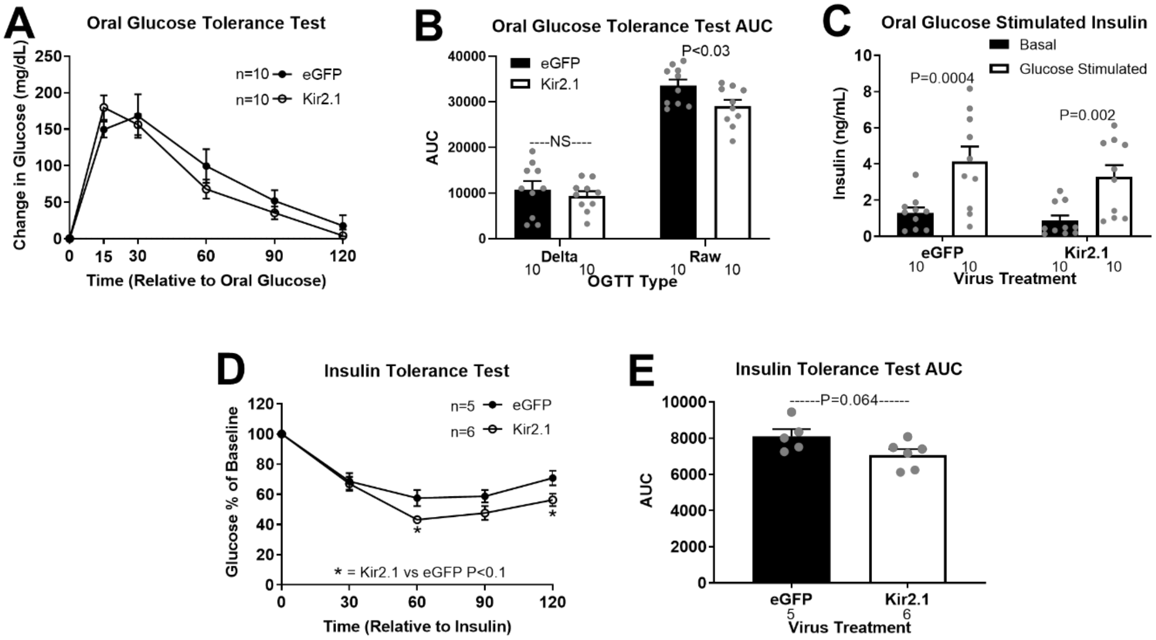

Figure S4. Related to Figure 3. Glucose homeostasis in Kir2.1 and eGFP control mice at 3 weeks of high fat diet feeding. Effect of hepatic Kir2.1 expression on oral glucose tolerance (OGTT; A), OGTT area under the curve (AUC; B), oral glucose stimulated serum insulin (C), insulin tolerance (ITT; D), and ITT AUC (E). NS = non-significant. Number below bar denotes n per group. All data are presented as mean ± SEM.

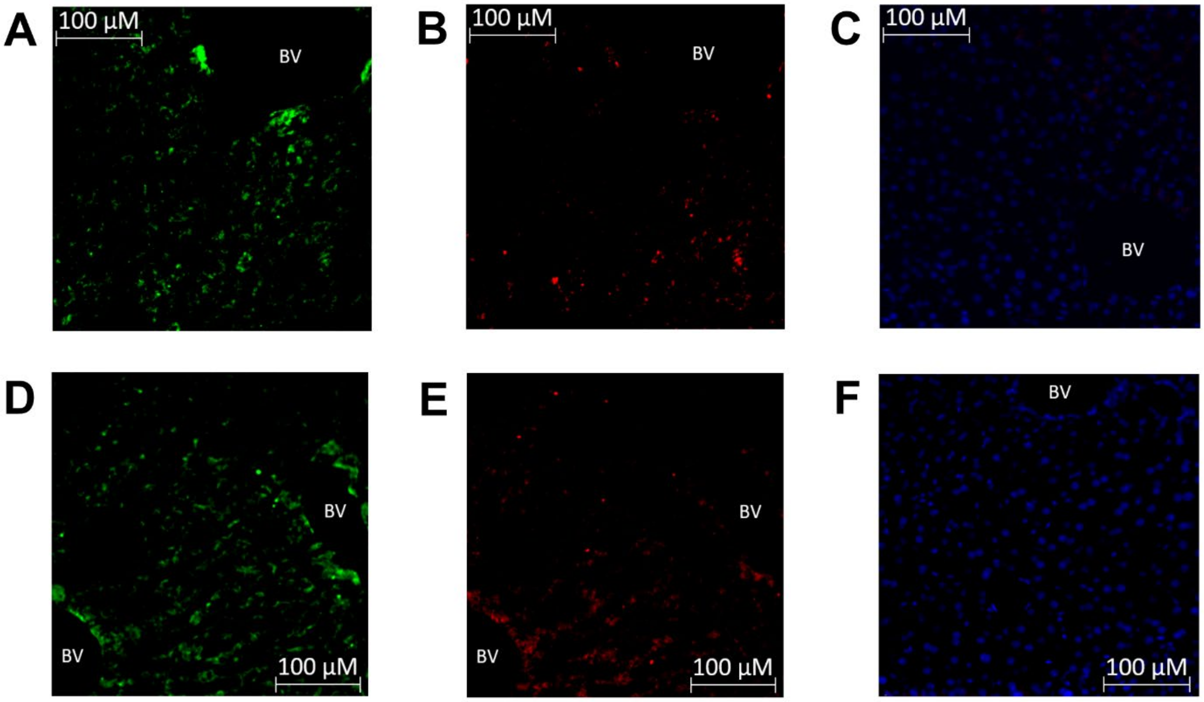

Figure S5. Related to Figure 6. Immunohistochemical evidence of $GABA_A$ receptor expressing vagal afferent innervation in the liver. Staining for the vagal afferent marker calretinin (A) and $GABA_A$ receptors (B) which correspond with the co-labeled image in Fig. 6A. No primary control imaged at the same settings as Fig. 6A (C). Staining for the alternative vagal afferent marker calcitonin gene-related peptide (CGRP; D) and $GABA_A$ receptors (E) which correspond with the co-labeled image in Fig. 6C. No primary control imaged at the same settings as Fig. 6C (F). Blue = DAPI (nucleus). Images at 10X magnification. BV = blood vessel

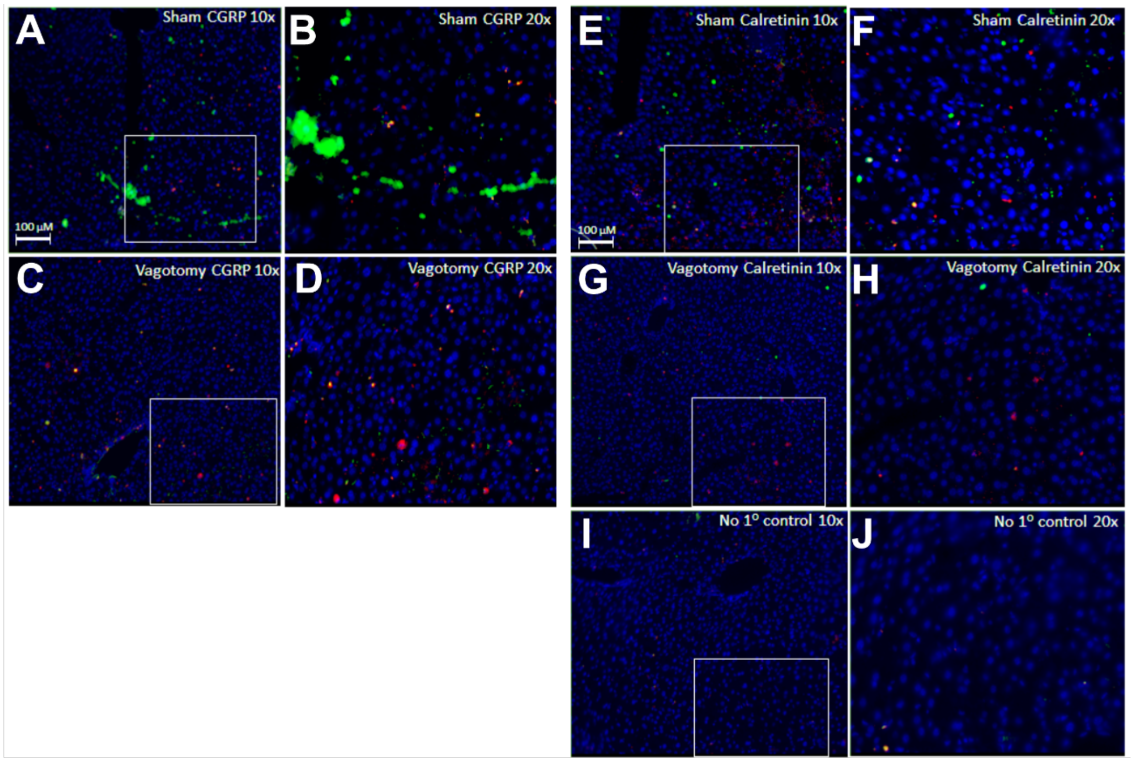

Figure S6. Related to Figure 6. Immunohistochemical evidence that hepatic vagotomy decreases immunohistochemical staining for 2 vagal afferent markers (green), calcitonin gene related peptide (CGRP; A-D) and calretinin (E-H). $GABA_A$ receptor is labeled in red. All images, including no-primary controls (I and J) were collected with identical settings. Blue = DAPI (nucleus). Images at 10 and 20X magnification as labelled.

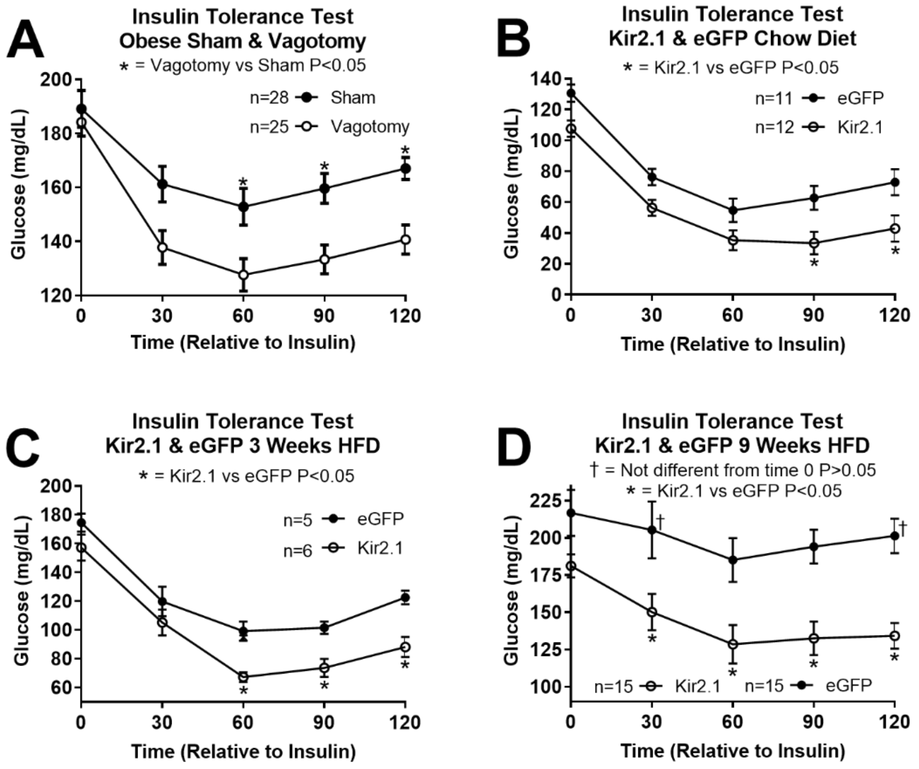

Figure S7. Related to Figures 1 and 3. Insulin tolerance tests (ITT) presented as raw glucose values. ITT in HFD fed sham and vagotomized mice (A). ITT in Kir2.1 and eGFP control mice on chow diet (B), and after 3 (C), and 9 weeks of HFD feeding (D). † Denotes the data point is not significantly different from time 0 for that group ($P > 0.05$). Unless indicated, all other timepoints are significantly different from time 0 within a group of mice. * Denotes significance between groups specified in the panel within a timepoint. All data are presented as mean ± SEM.

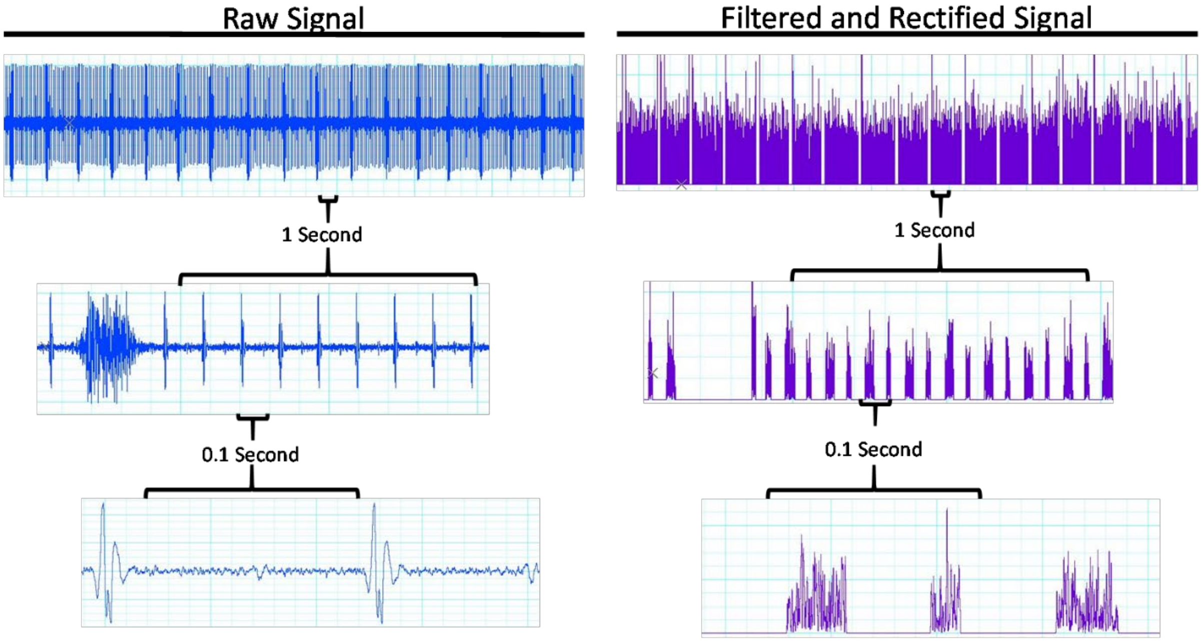

Figure S8. Related to Figure 2. Raw signal generated from vagal nerve recordings including ECG signal and breathing artifacts. Data was filtered to remove these signals that are not specific to vagal nerve activity and the signal rectified to allow for integration of total nerve bundle activity. Top to bottom includes more zoomed in versions of the timeline to allow the reader to understand exactly what was analyzed.

Table S1. Liver slice neurotransmitter panel data. Related to Figure 4.

Neurotransmitter (µmol/µg DNA)	Lean (N = 5)	Obese (N = 3)	% Change in Obesity
**Adenosine**	0.22 ±0.04	0.10 ± 0.01	-55%*
**Histidine**	17.74 ± 0.92	12.90 ± 0.72	-27%*
**Serine**	22.32 ± 3.33	13.02 ± 0.53	-42%
**Taurine**	238.40 ± 18.41	305.18 ± 38.04	28%
**Glutamine**	49.06 ± 5.19	40.39 ± 3.98	-17%
**Glycine**	130.74 ± 5.16	81.31 ± 4.93	-37%*
**Aspartic Acid**	6.92 ± 0.55	3.47 ± 0.32	-50%*
**Glutamic Acid**	30.32 ± 2.12	28.74 ± 3.48	-5.2%
**GABA**	5.43 ± 0.64	8.77 ± 0.53	61%*

Initial neuromodulators panel analysis on media collected form the liver explant studies performed by the Mayo Clinic Metabolomics Regional Core. *Indicates significant difference between obese and lean mice ($P<0.05$). Data are presented as mean ± SEM.

Table S2. Metabolic characteristics of the study subjects (n=19). Related to Figure 7.

	Mean ± SEM	Range
Body mass index (kg/m^2)	45.1 ± 1.3	35.9 - 55.6
Intrahepatic triglyceride content (%)	11.4 ± 1.9	2.7 - 28.0
Glucose (mg/dL)	97 ± 2	81 - 121
Insulin (μU/mL)	24.1 ± 1.7	13.1 - 46.5
Glucose infusion rate during insulin infusion (μmol/kg FFM/min)	36.0 ± 3.0	15.2 - 60.8
Glucose Rd during insulin infusion (% increase)	131 ± 19	30 - 355

FFM, fat free mass; Glucose Rd, glucose disposal rate.

Table S3. Related to Figure 7. Regression coefficient estimates showing the association between hepatic mRNA expression of genes involved in GABA production (ABAT) and GABA transport (Slc6A6, A8, A12, and A13) and glucose infusion rate (μMol/Kg Fat Free Mass/min) and Glucose Rd (rate of disposal; % increase) during a hyperinsulinemic-euglycemic clamp.

Glucose Infusion Rate (μMol/Kg Fat Free Mass/min)					
	**Estimate**	**SEM**	**Lower CI**	**Upper CI**	**P- Value**
**Intercept**	-36.41	53.75	-158.00	85.18	0.5152
**IHTG (%)**	-0.50	0.19	-0.92	-0.08	0.0242
**SLC6A12**	13.80	5.72	0.86	26.74	0.0391
**SLC6A13**	10.74	4.18	1.28	20.20	0.0302
**SLC6A6**	-15.63	3.20	-22.87	-8.38	0.0009
**SLC6A8**	-5.65	1.95	-10.07	-1.23	0.0179
**ABAT**	-3.26	7.04	-19.20	12.67	0.6545
**Glucose Rd During Insulin Infusion (% Increase)**					
	**Estimate**	**SEM**	**Lower CI**	**Upper CI**	**P- Value**
**Intercept**	-3.91	4.24	-13.49	5.68	0.3805
**IHTG (%)**	-0.03	0.01	-0.07	0.00	0.0427
**SLC6A12**	1.02	0.45	0.00	2.04	0.0505
**SLC6A13**	0.64	0.33	-0.10	1.39	0.0834
**SLC6A6**	-0.71	0.25	-1.28	-0.14	0.0204
**SLC6A8**	-0.45	0.15	-0.79	-0.10	0.0178
**ABAT**	-0.24	0.56	-1.50	1.01	0.6712

Table S4. Related to Figure 7. Single nucleotide polymorphisms (SNPs) that result in missense mutations in GABA transporters are associated with an increased incidence (OR; odds ratio) of type 2 diabetes (T2D; source: knowledge portal diabetes database). MAF – minor allele frequency.

***SLC6A12* - T2D Associated SNPs**							
**Variant ID**	**dbSNP ID**	**Predicted Impact**	**Study**	**P-value**	**Effect**	**OR**	**MAF**
12_313839_G_A	rs188610	Missense: synonymous variant	AMP T2D-GENES T2D exome sequence analysis	0.0238	↑	1.1	0.0386
12_313824_G_A	rs199521597	Missense: early stop codon	BioMe AMP T2D GWAS	0.0409	↑	15.8	0.000269
***SLC6A13* - T2D Associated SNPs**							
**Variant ID**	**dbSNP ID**	**Predicted Impact**	**Study**	**P-value**	**Effect**	**OR**	**MAF**
12_330193_C_T	rs61741313	Missense: Replaces Arganine with Glutamine	DIAMANTE (European) T2D GWAS	0.04	↑	1.04	0.01291-0.0531
***SLC6A6* -T2D Associated SNPs**							
**Variant ID**	**dbSNP ID**	**Predicted Impact**	**Study**	**P-value**	**Effect**	**OR**	**MAF**
3_14489107_G_A	rs62233560	Missense: Replaces Valine with Isoleucine	AMP T2D-GENES T2D exome sequence analysis: Europeans	0.00143	↑	1.4	0.005-0.0165
3_14523296_G_A	rs41284017	Missense: Replaces Valine with Isoleucine	70KforT2D GWAS	0.00234	↑	1.38	0.0062-0.0165
***SLC6A12* - T2D Adjusted for BMI Associated SNPs**							
**Variant ID**	**dbSNP ID**	**Predicted Impact**	**Study**	**P-value**	**Effect**	**OR**	**MAF**
12_302492_C_G	rs138178078	Missense: Replace Tryptophan with Serine	ExTexT2D exome chip analysis	0.000071	↑	1.26	0.0052
12_319125_A_G	rs557881	Missense: Replace Cysteine with Arginine	ExTexT2D exome chip analysis	0.0396	↑	1.01	0.48
12_300248_C_G,T	rs147574089	Missense: Replace Glutamate with Glutamine	CAMP GWAS	0.0397	↑	6.09	0.0012
***SLC6A13* - T2D Associated SNPs**							
**Variant ID**	**dbSNP ID**	**Predicted Impact**	**Study**	**P-value**	**Effect**	**OR**	**MAF**
12_346454_C_T	rs140951084	Missense in Splice Region: Replace Arginine with Glutamine	BioMe AMP T2D GWAS	0.0359	↑	3.55	0.0019
